# RBPJ, the Major Transcriptional Effector of Notch Signaling, Remains Associated with Chromatin throughout Mitosis, Suggesting a Role in Mitotic Bookmarking

**DOI:** 10.1371/journal.pgen.1004204

**Published:** 2014-03-06

**Authors:** Robert J. Lake, Pei-Fang Tsai, Inchan Choi, Kyoung-Jae Won, Hua-Ying Fan

**Affiliations:** 1 Epigenetics Program, Department of Biochemistry and Biophysics, University of Pennsylvania, Philadelphia, Pennsylvania, United States of America; 2 Institute for Diabetes Obesity and Metabolism, University of Pennsylvania, Philadelphia, Pennsylvania, United States of America; 3 Department of Genetics, Perelman School of Medicine, University of Pennsylvania, Philadelphia, Pennsylvania, United States of America; Thomas Jefferson University, United States of America

## Abstract

Mechanisms that maintain transcriptional memory through cell division are important to maintain cell identity, and sequence-specific transcription factors that remain associated with mitotic chromatin are emerging as key players in transcriptional memory propagation. Here, we show that the major transcriptional effector of Notch signaling, RBPJ, is retained on mitotic chromatin, and that this mitotic chromatin association is mediated through the direct association of RBPJ with DNA. We further demonstrate that RBPJ binds directly to nucleosomal DNA *in vitro*, with a preference for sites close to the entry/exit position of the nucleosomal DNA. Genome-wide analysis in the murine embryonal-carcinoma cell line F9 revealed that roughly 60% of the sites occupied by RBPJ in asynchronous cells were also occupied in mitotic cells. Among them, we found that a fraction of RBPJ occupancy sites shifted between interphase and mitosis, suggesting that RBPJ can be retained on mitotic chromatin by sliding on DNA rather than disengaging from chromatin during mitotic chromatin condensation. We propose that RBPJ can function as a mitotic bookmark, marking genes for efficient transcriptional activation or repression upon mitotic exit. Strikingly, we found that sites of RBPJ occupancy were enriched for CTCF-binding motifs in addition to RBPJ-binding motifs, and that RBPJ and CTCF interact. Given that CTCF regulates transcription and bridges long-range chromatin interactions, our results raise the intriguing hypothesis that by collaborating with CTCF, RBPJ may participate in establishing chromatin domains and/or long-range chromatin interactions that could be propagated through cell division to maintain gene expression programs.

## Author Summary

How does a cell remember what it should be after cell division? One mechanism that is beginning to emerge is the retention of a few key regulatory proteins on the highly condensed mitotic chromatin during cell division. These proteins are called mitotic bookmarks, as they are believed to offer critical information as to how genetic information should be read immediately after mitosis. We have found that a protein called RBPJ, which plays pivotal roles in regulating cell-fate choices, is retained on mitotic chromatin. RBPJ transmits to DNA signals elicited by the Notch pathway: a pathway that conveys information resulting from the communication between two adjacent cells. Unlike many other factors, we found that RBPJ can bind to nucleosomes, which are the basic unit of packaged DNA consisting of DNA wrapped around eight histone proteins. We also found that RBPJ interacts with and binds to DNA sites regulated by the CTCF protein, which plays important roles in regulating long-range DNA interactions. Together, our results suggest that RBPJ can function as a mitotic bookmarking factor, to help maintain genetic programs, higher-order structural information and consequently the memory of cell identity through cell division.

## Introduction

The faithful propagation of transcriptional programs through mitosis is important to maintain cell identity. During mitosis, DNA becomes highly condensed, transcription ceases and many key regulatory proteins, such as transcription factors, enhancer-binding proteins and RNA polymerases, disengage from mitotic chromatin. However, the memory of a specific gene expression program that is required to maintain cell identity mysteriously persists. How then is transcriptional memory maintained through mitosis?

Several different mechanisms have been proposed to control the maintenance of transcriptional memory through mitosis [1]. DNA methylation can be used to propagate repressed chromatin states. Histone modifications and histone variants are also believed to be important marks to convey the signatures of active and repressed genes to progeny cells [2]. For instance, the maintenance of methylation on histone H3 lysine 9 during mitosis may be important for the recruitment of HP1 (heterochromatin protein 1) upon mitotic exit and, consequently, the reestablishment of heterochromatin domains. Histone marks that persist through mitosis can also mediate the retention of histone code readers on mitotic chromatin, such as the retention of Brd4 (Bromodomain-containing protein 4) by acetylated histone H4, which may be important to maintain decondensed chromatin regions and the rapid recruitment of transcription factors in G1 [3]. Moreover, a shifting of the histone variant H2A.Z from the +1 position upstream to the transcription start site may help to temporarily repress the transcription of genes that are otherwise marked as active [4].

Interestingly, there also appears to be mechanisms to propagate long-range chromosomal interactions through cell division. For example, the CTCF protein (CCCTC-binding factor) is a sequence specific DNA-binding factor that can function as a transcription activator, repressor or insulating factor, depending on the context [5]. CTCF also functions in X-chromosome inactivation as well as in the allele-specific expression of imprinted loci through mediating the formation of long-range chromosomal interactions. Evidence suggests that the sequence-specific DNA binding of CTCF and the long-range chromosomal interactions mediated by CTCF may be maintained through mitosis [6]. Moreover, PSC, a Drosophila polycomb group (PcG) protein, has recently been found to be retained at specific regions of mitotic chromatin, and this mitotic chromatin-association likely facilitates the efficient reestablishment of PcG function and specific long-range chromatin interactions upon mitotic exit [7].

Accumulating evidence indicates that the maintenance of transcriptional memory through mitosis is also achieved through the epigenetic marking of genes by select transcription factors, a process often referred to as mitotic bookmarking [1]. Mitotic bookmarking may help define the kinetics of transcription activation or repression upon mitotic exit [8]. Although the mechanism by which bookmarks impact transcription kinetics is largely unknown, these transcription factors may maintain regions of open chromatin for the rapid recruitment of the transcriptional machinery upon mitotic exit [1]. Importantly, lineage specific transcription factors, such as GATA1 and FOXA1, are emerging as key players in mitotic bookmarking [9], [10].

In this study, we demonstrate that the transcription factor RBPJ, which participates in both transcriptional activation and repression, possesses properties of a mitotic bookmarking factor. RBPJ is the major downstream effector of the evolutionarily conserved Notch signaling pathway [11]–[16], a pathway that is critical to numerous developmental processes that range from stem cell maintenance to neurogenesis. Our study offers novel insights into the mechanisms by which RBPJ and, consequently, Notch signaling can maintain transcriptional memory and cell-fate choices through cell division.

## Results

### RBPJ associates with mitotic chromatin

To identify transcription factors that can function as epigenetic marks, we purified mitotic chromatin from the murine embryonal carcinoma cell line F9, which is believed to represent the cancer stem cell of a teratocarcinoma [17]. Proteins extracted from mitotic chromatin were subjected to mass spectrometric analysis. We found that the transcription factor and major downstream effector of Notch signaling, RBPJ, was associated with mitotic chromatin.

**Figure 1:**
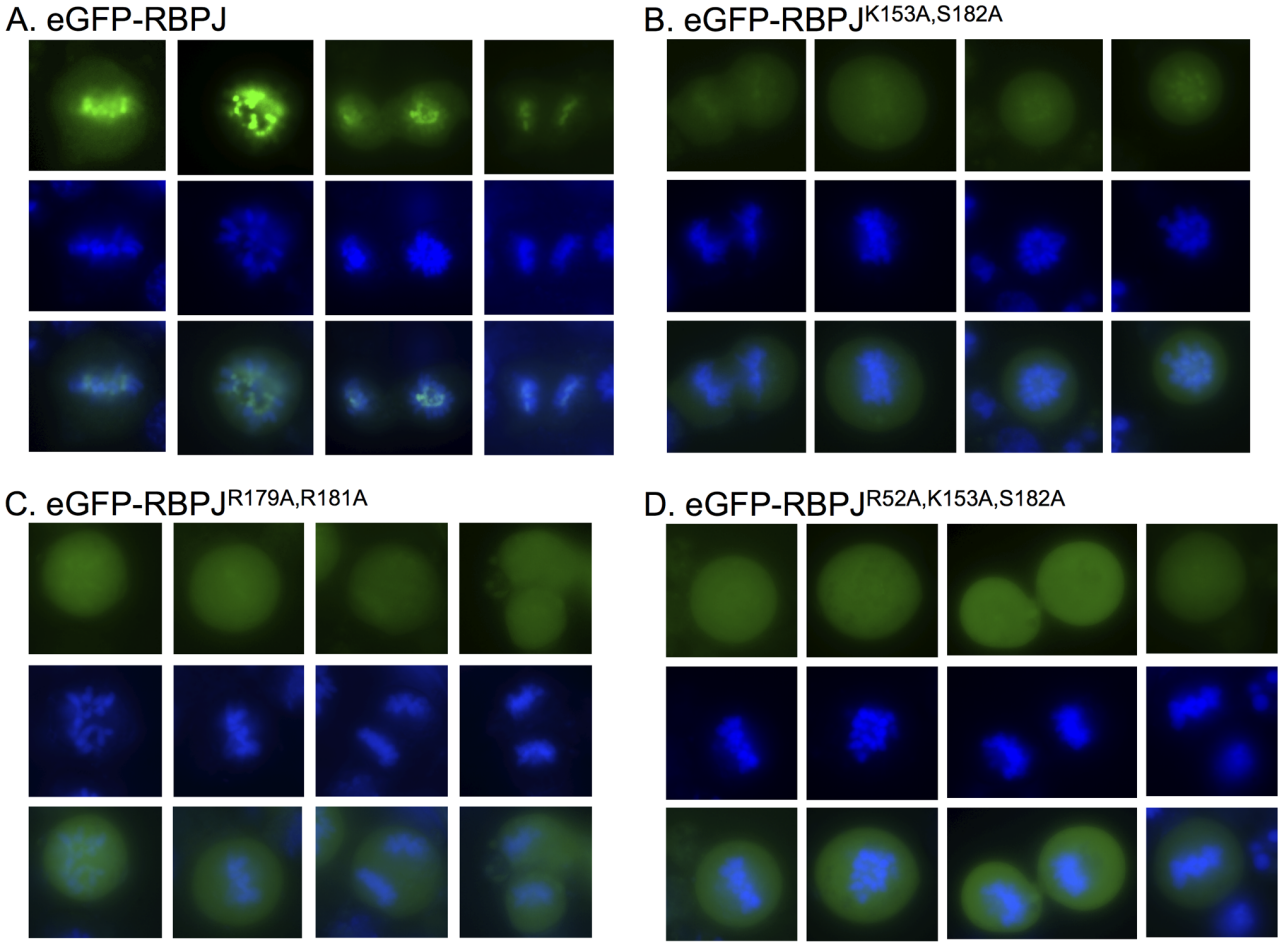
RBPJ associates with mitotic chromatin through direct DNA contacts. Fluorescence microscopy imaging of live F9 cells transiently expressing eGFP-tagged RBPJ or RBPJ derivatives harboring mutations in residues predicted to contact DNA (green). Chromatin was counterstained with Hoechst 33342 (blue). (A) F9 cells expressing eGFP-RBPJ. (B) F9 cells expressing eGFP-RBPJ^K153A,S182A^. (C) F9 cells expressing eGFP-RBPJ^R179A,R181A^. (D) F9 cells expressing eGFP-RBPJ^R52A,K153A,S182A^. R52A, K153 and S182 are conserved residues predicted to contact DNA bases. R179 and R181 are conserved residues predicted to contact the phosphodiester backbone.

To confirm that RBPJ was retained on mitotic chromatin, we expressed an eGFP-tagged derivative of RBPJ in F9 cells. As shown in Figure 1A, fluorescence imaging of live F9 cells revealed that eGFP-RBPJ remained associated with mitotic chromatin. Additionally, when compared to the mitotic chromatin signal, as revealed by counterstaining with Hoechst 33342, the eGFP-RBPJ signal appeared more restricted, suggesting that eGFP-RBPJ is enriched at specific regions of mitotic chromatin in F9 cells (Figures 1A and S1).

### Conserved RBPJ residues that directly contact DNA contribute to mitotic chromatin retention

RBPJ is a sequence-specific DNA binding protein that binds with high affinity to the consensus sequence TTCCCAC(A/G) [18]. We next determined if the retention of RBPJ on mitotic chromatin was mediated through its direct interaction with DNA, or whether it was mediated through an indirect association with another component of mitotic chromatin. Crystal structure studies have revealed conserved residues of RBPJ that contact DNA [19], [20]. To understand the mechanism by which RBPJ associates with mitotic chromatin, we introduced mutations into RBPJ that are predicted to interfere with either DNA base (K153A, S182A and R52A) or phosphodiester (R179A and R181A) interactions. Fluorescence imaging of live F9 cells revealed that, in all cases, the mutations decreased the retention of RBPJ on mitotic chromatin. We found that the association of eGFP-RBPJ^K153A, S182A^ and eGFP-RBPJ^R179A, R181A^ with mitotic chromatin was significantly reduced (Figures 1B–C and S1), and that the association of eGFP-RBPJ^R52A,^ ^K153A, S182A^ was reduced even further (Figures 1D and S1). However, residual mitotic chromatin association of RBPJ was always maintained, as a complete loss of chromatin association would have resulted in the appearance of negative chromatin staining [21]. Additionally, the residual mitotic chromatin association of the RBPJ derivatives did not display the more restricted chromatin-staining pattern observed with wild-type RBPJ, suggesting loss of binding specificity. From this analysis, we conclude that the association of RBPJ with mitotic chromatin is mediated through direct contacts with DNA, and that conserved RBPJ residues that contact both DNA bases and the phosphodiester backbone contribute to this association.

### RBPJ binds to nucleosomal DNA *in vitro*

**Figure 2:**
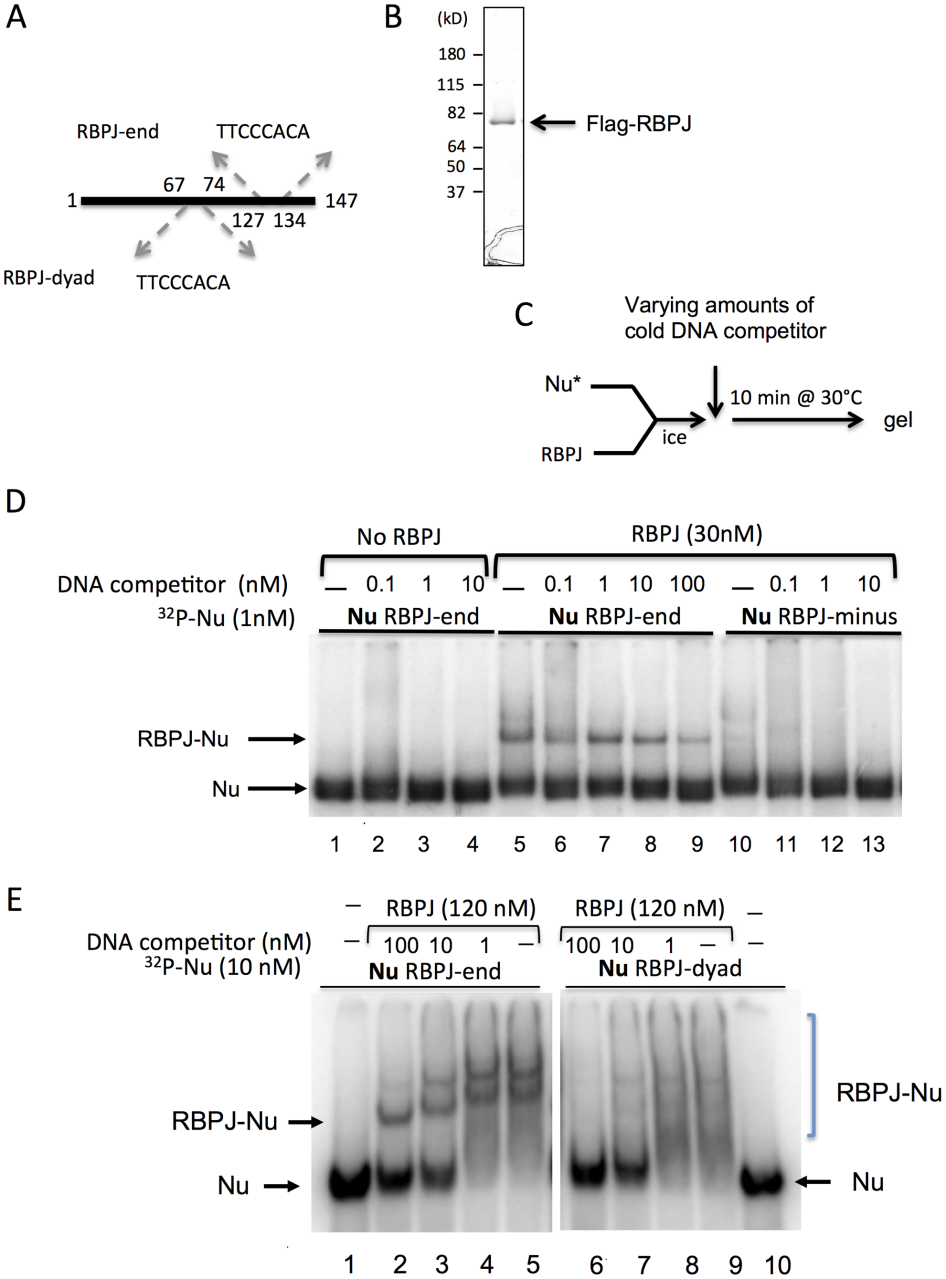
RBPJ interacts with core mononucleosomes. (A) Three 147 bp DNA fragments were used to assemble nucleosomes for gel shift assays. All DNA fragments contain nucleosome-phasing sequences between positions 1–40 [49]. RBPJ-end and RBPJ-dyad contain an RBPJ-binding motif between positions 127–134 and 67–74, respectively. The RBPJ-minus fragment does not contain an RBPJ-binding motif. (B) Recombinant Flag-tagged RBPJ protein purified from insect SF9 cells and used in assays shown in D–E. (C) Experimental scheme. (D) ^32^P-labeled nucleosomes (Nu) were used at 1 nM. RBPJ was used at 30 nM. 147 bp DNA (RBPJ-minus) was used as cold competitor at final concentrations of 0.1, 1, 10 nM (lanes 2–4, 6–8, and 11–13, respectively). 100 nM cold-competitor DNA was used in lane 9, to emphasize the stability of the RBPJ-nucleosome complex. (E) Two mononucleosome cores were used in the binding assays: RBPJ-dyad and RBPJ-end nucleosomes, containing the RBPJ binding motifs close to the dyad and entry/exit position, respectively. ^32^P-labeled nucleosomes (Nu) were used at 10 nM. RBPJ was used at 120 nM. 147 bp DNA (RBPJ-minus) was used as cold-competitor DNA at final concentrations of 100, 10 and 1 nM to demonstrate that the RBPJ protein specifically interacted with the RBPJ-binding motif (indicated by an arrow). RBPJ-nucleosome complexes of different stoichiometries are indicated by a bracket.

Nucleosomes are the basic unit of chromatin, consisting of approximately 147 bp of DNA wrapped around an octamer of histone proteins, and nucleosomes have been shown to interfere with the binding of many transcription factors to DNA. Given that the DNA of mitotic chromatin is highly condensed, we determined if RBPJ had the capacity to directly associate with nucleosomal DNA. To accomplish this, Flag-tagged RBPJ was purified from SF9 cells and used in electrophoretic mobility shift assays (Figure 2). Three, 147 bp DNA fragments were used (Figures 2A and S2A): (1) a fragment without an RBPJ-binding motif (RBPJ-minus), (2) a fragment with an 8 bp RBPJ-binding motif (TTCCCACA) positioned 13 nucleotides from one of the ends (RBPJ-end), and (3) a fragment containing a binding motif located close to the fragment's center (RBPJ-dyad). These different DNA fragments would allow us to determine if RBPJ could bind nucleosomal DNA, and whether the translational position of a binding motif on the surface of a nucleosome could impact the association of RBPJ with nucleosomal DNA.

As expected, purified RBPJ (Figure 2B) interacted specifically with a 147 bp naked DNA fragment harboring an 8-bp RBPJ-binding motif (Figure S2C, lanes 4 and 5). Moreover, this sequence-dependent RBPJ-DNA interaction persisted even in the presence of 100-fold molar excess of competitor DNA (Figure S2C, lane 5).

We next asked if the RBPJ protein could interact with its binding motif in the context of nucleosomal DNA (Figure 2). We first examined the binding of purified RBPJ to core nucleosomes assembled with the RBPJ-minus and RBPJ-end DNA fragments (Figures 2 and S3).

As shown in Figure 2D (lanes 5–9), RBPJ was able to associate with nucleosomal DNA harboring an RBPJ-binding motif, even in the presence 100-fold molar excess of competitor DNA. The shifts in nucleosome mobility were due to RBPJ binding, as mobility-shifted bands did not appear in the absence of the RBPJ protein. These results demonstrated that RBPJ could directly bind to nucleosomal DNA, in contrast to most other transcription factors [1].

We next compared the binding of RBPJ to nucleosomes containing the RBPJ-binding motif positioned close to the DNA entry/exit site (RBPJ-end) to that of nucleosomes containing the binding motif close to the nucleosome center (RBPJ-dyad). As shown in Figure 2E, while a clear preference for RBPJ binding to its cognate motif positioned close to the DNA entry/exit sites could be observed (lanes 2–5), little or no discrete binding of RBPJ to nucleosomes containing its cognate motif positioned close to the nucleosome dyad was detected (lanes 7–9). Taken together, these results revealed that RBPJ can bind to nucleosomal DNA with higher affinity when the RBPJ-binding motif is positioned closer to the DNA entry/exit site.

### Sites of RBPJ occupancy in asynchronous and mitotic F9 cells

We next compared the sites of RBPJ occupancy at a genome-wide level on interphase and mitotic chromatin, by performing anti-RBPJ chromatin immunoprecipitation experiments followed by deep sequencing (ChIP-seq). To perform these analyses, we generated an anti-RBPJ antibody, using a GST-RBPJ fusion protein purified from insect SF9 cells. To examine the specificity of the resulting antibody, we reduced RBPJ protein levels in F9 cells using shRNA-mediated RNA interference. As shown in Figure S4A, the anti-RBPJ antibody recognized a protein of approximately 55 kD in lysates prepared from F9 cells expressing a control shRNA. Moreover, the level of the protein recognized by the antibody was substantially diminished in lysates prepared from F9 cells expressing an shRNA targeting RBPJ. Significantly, immunoprecipitation of RBPJ from crosslinked 293T cells followed by western blot analysis demonstrated that the RBPJ antibody could be used in ChIP assays (Figure S4B).

To identify sites of RBPJ occupancy on interphase and mitotic chromatin, we performed ChIP, using the anti-RBPJ antibody, on asynchronous (cycling) and mitotic (nocodazole-arrested) F9 cells. The mitotic F9 cells used for ChIP studies were generally more than 98% pure (Figure S5). The resulting immunoprecipitated DNA was made into a library for deep sequencing. The sequencing reads were mapped to the mouse genome using the Bowtie aligner [22]. Peak calling was carried out using HOMER (Hypergeometric Optimization of Motif EnRichment) with a default option (FDR = 0.001 and Poisson p-value cutoff = 0.0001) on ChIPed samples against the matching input samples, and then a 1 RPM cutoff was applied [23]. Specific peaks were defined as having at least a four-fold difference in enrichment within a 200 bp region between the two cell populations and a Poisson enrichment p-value less than 0.0001. The remaining peaks were defined as common peaks.

**Figure 3:**
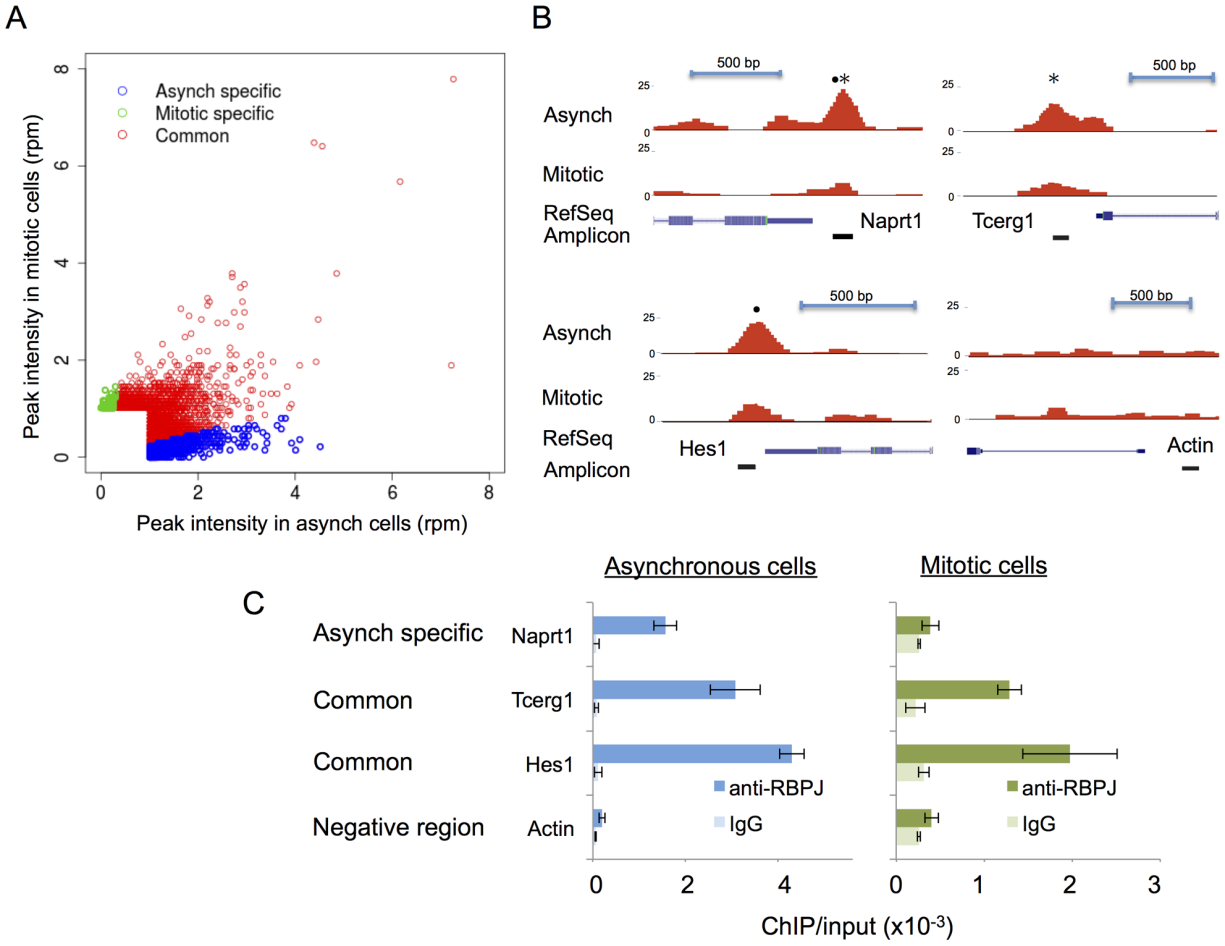
The majority of RBPJ occupancy sites are common in mitotic and asynchronous cells. (A) Scatter plot showing the correlation of RBPJ occupancy sites on asynchronous and mitotic chromatin (rpm: reads per million). Asynchronous-specific, mitotic-specific and common occupancy sites are as noted. (B) Screen shots taken from the UCSC genome browser showing RBPJ-binding peaks at three genomic loci as indicated. RBPJ occupies the Naprt1 promoter in asynchronous cells, while RBPJ occupies the Hes1 and Tecrg1 promoters in both asynchronous and mitotic cells. A region of the actin gene was used as a negative control. The relative positions of RBPJ-binding motifs (•) and CTCF-binding motifs (*) within the peaks are as indicated. (C) Validation of the ChIP-seq results using ChIP-qPCR.

Using these criteria, we identified 1851 asynchronous-specific peaks, 545 mitotic-specific peaks and 2736 common peaks (Table 1). As shown in Figure 3A, ∼40% of the RBPJ occupancy sites in asynchronous cells were unique to asynchronous cells (asynchronous specific, blue), and ∼20% of RBPJ occupancy sites in mitotic cells were unique to mitotic cells (mitotic specific, green). Approximately, 60% of the RBPJ occupancy sites in asynchronous cells were retained on mitotic chromatin, which accounts for ∼80% of the total RBPJ occupancy sites in mitotic cells (common, red). These common peaks likely cover genomic regions subjected to bookmarking by RBPJ (Table S1).

**Table 1:** Summary of anti-RBPJ ChIP-seq results.

	Number of Peaks	RBPJ motif	CTCF motif	RBPJ or CTCF	RBPJ and CTCF
Asynchronous Specific^1^	1851	381 (21%)	255 (14%)	596 (32%)	40 (2%)
Mitotic Specific^1^	545	26 (5%)	9 (2%)	33 (6%)	2 (0.4%)
Common^2^	2736	382 (14%)	490 (18%)	800 (29%)	72 (3%)

1 A specific peak was defined as having enrichment within a 200 bp region of more than 4-fold between the two cell populations and a Poisson enrichment p-value<0.0001.

**Figure 4:**
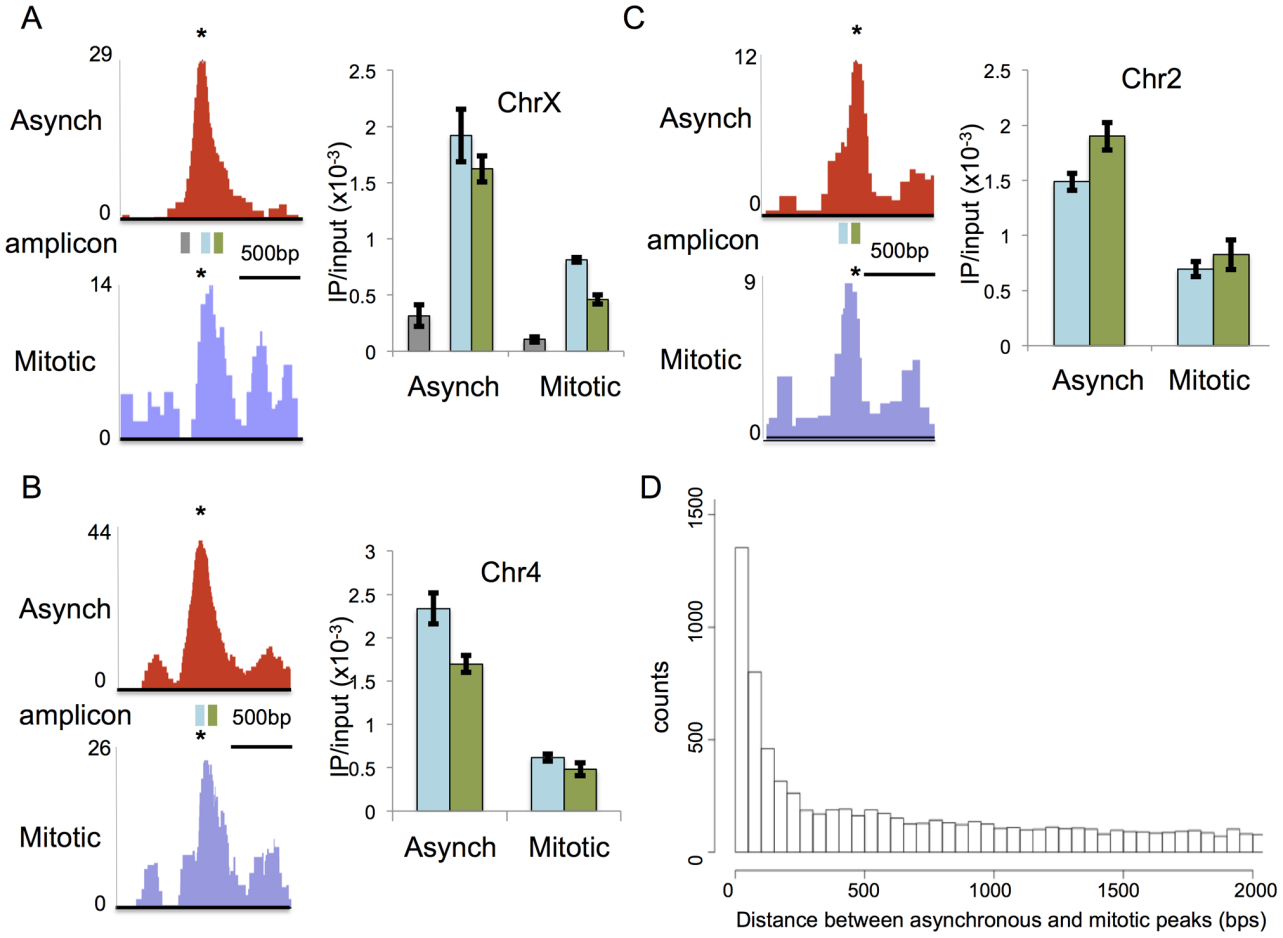
RBPJ occupancy sites can shift between interphase and mitosis. Left panels in (A–C) are screen shots taken from the UCSC genome browser showing RBPJ-binding peaks from asynchronous (red) and mitotic (blue) cells at three sites of RBPJ occupancy: (A) chrX:131,119,512-131,120,980, (B) chr4:118,743,747-118,745,201 and (C) chr2:181,230,559-181,231,788. The position of the RBPJ-binding motif within each peak is indicated with an asterisk. Right panels in (A–C) are results from ChIP-qPCR assays using two primer sets that span the asynchronous and mitotic peaks. The positions of the amplicons are noted by rectangles underneath the peaks (blue and green). The gray amplicon in (A) was used as a negative control region. The ChIP-qPCR results confirmed shifts in the centers of the RBPJ-binding peaks between asynchronous and mitotic cells at these loci. Primers used are described in Table S6. (D) Histogram showing the number of peaks as a function of separation distance between asynchronous and mitotic cells. Bin size is 50 bps.

2 A common peak was defined as having similar enrichment between mitotic and asynchronous peaks. As we observed peak shifting (see Figure 4), common peaks were centered on asynchronous peaks. If centered on mitotic peaks, 8.2% and 8.4% of common peaks contain RBPJ and CTCF motifs, respectively.

ChIP-qPCR was used to validate the cell cycle-dependent association of RBPJ with sites in the promoters of three genes (Figure 3B): Naprt1 (Nicotinate phosphoribosyltransferase), Tcerg1 (Transcription elongation regulator 1), and the known RBPJ/Notch target Hes1 (Hairy and enhancer of split 1). Of the three regions analyzed, the Hes1 promoter contained an RBPJ-binding motif (•), the Tcerg1 promoter contained a CTCF-binding motif (*) and the Naprt1 promoter contained both binding motifs (see below for motif analysis of RBPJ occupancy sites). As shown in Figure 3C, ChIP-qPCR revealed that the association of RBPJ with these three promoters mirrored the association as determined by ChIP-seq. RBPJ occupied the Tcerg1 and Hes1 promoters in both asynchronous and mitotic cells, but RBPJ preferentially occupied the Naprt1 promoter in asynchronous cells. We did not observe RBPJ association with a region of the actin gene, which was used as a negative control.

### Genomic annotation and gene ontology of RBPJ occupancy sites

Between each of these three peak categories (asynchronous specific, mitotic specific and common), genomic annotation revealed little difference in the relative percentages for most functional genomic classes (Figure S6 and Tables S2, S3): intergenic regions (∼35–45%), introns (∼28–43%), promoters (16–27%), exons (∼6%), transcription start sites (∼2%), 3′ UTRs (∼1%) and 5′ UTRs (∼1%). Of interest, when we compared RBPJ occupancy at sites that specifically contained RBPJ-binding motifs, we found that the percentage of mitotic-specific promoter occupancy was relatively low (3.6%) compared to asynchronous-specific promoter occupancy (21.5%).

To categorize further the functions of genes near sites of RBPJ occupancy, gene ontology (GO) analysis was performed with the Genomic Regions Enrichment of Annotations Tool (GREAT) (Tables S4, S5) [24]. Genes involved in stem cell maintenance, development and differentiation-related pathways correlated most significantly with sites of RBPJ occupancy in asynchronous F9 cells (Table S4). These results agree with the known functions of RBPJ as the major transcriptional effector of the Notch signaling pathway [12]. GO analysis also suggests an involvement of RBPJ in the metabolism and processing of non-coding RNAs, consistent with the known functions of RBPJ/Notch in miRNA biogenesis. Moreover, results from the GO analysis suggest that these properties may extend to small nuclear and nucleolar RNA, telomerase RNA components and Cajal body-specific RNA (Tables S4) [25].

### A fraction of RBPJ occupancy sites shift between mitotic and asynchronous cells

During the course of our analysis, we found that some of the peaks obtained by ChIP-seq appeared to shift in their genomic localization between mitotic and asynchronous cells (Figure 4). This was revealed by changes in the position of the RBPJ-binding motifs relative to the peak centers. To validate this observation, we analyzed RBPJ enrichment at three different loci that showed peak shifting, using ChIP-qPCR with primer sets positioned around the different peaks. All three loci contained RBPJ-binding motifs (*) at their peak centers in asynchronous F9 cells but not at the peak centers in mitotic cells (Figure 4A–C, left panels). If the positions of RBPJ peaks of asynchronous cells were the same as that of mitotic cells, we would expect to see a similar ratio of DNA enrichment using two adjacent primer sets. As shown in Figure 4A–C (right panels), we observed changes in the ratios of DNA amplified by different primer sets (blue vs. green) when we compared asynchronous and mitotic chromatin isolated by RBPJ-ChIP. Specifically, for the chrX locus, similar amounts of DNA were amplified by both primer sets from asynchronous chromatin (p-value = 0.3) but different amounts from mitotic chromatin (p-value = 0.002) (Figure 4A). At the chr2 and chr4 loci, similar amounts of DNA were amplified by both primer sets from mitotic chromatin (p = 0.2 and 0.4, respectively) but different amounts from asynchronous chromatin (p = 0.04 and 0.05, respectively) (Figure 4B–C). Importantly, all of the qPCR signals were significantly higher than that of a negative control region (Figure 4A, gray). Together, these results support the hypothesis that RBPJ can shift away from some of its preferred interphase binding sites during mitosis.

Intrigued by these observations, we increased the depth of our analysis by calculating the distances between all neighboring RBPJ peaks in asynchronous and mitotic cells, and then plotting the number of peaks as a function of separation distance. As shown in Figure 4D, there was a tendency to find differences in peak positions between asynchronous and mitotic cells with a distance of 250 bp or less, and this tendency increased as separation distance decreased. Some of these peaks represent common sites of RBPJ occupancy, but with shifts in their translational position between asynchronous and mitotic cells. Taken together, these results support the notion that, during mitosis, RBPJ can shift away from its preferred DNA-binding site (Figure 4). Given that RBPJ can be retained on mitotic chromatin, these results also suggest that RBPJ can slide along DNA when chromatin is restructured during mitosis, to maintain its local chromatin association.

### RBPJ- and CTCF-binding motifs are enriched at sites of RBPJ occupancy

**Figure 5:**
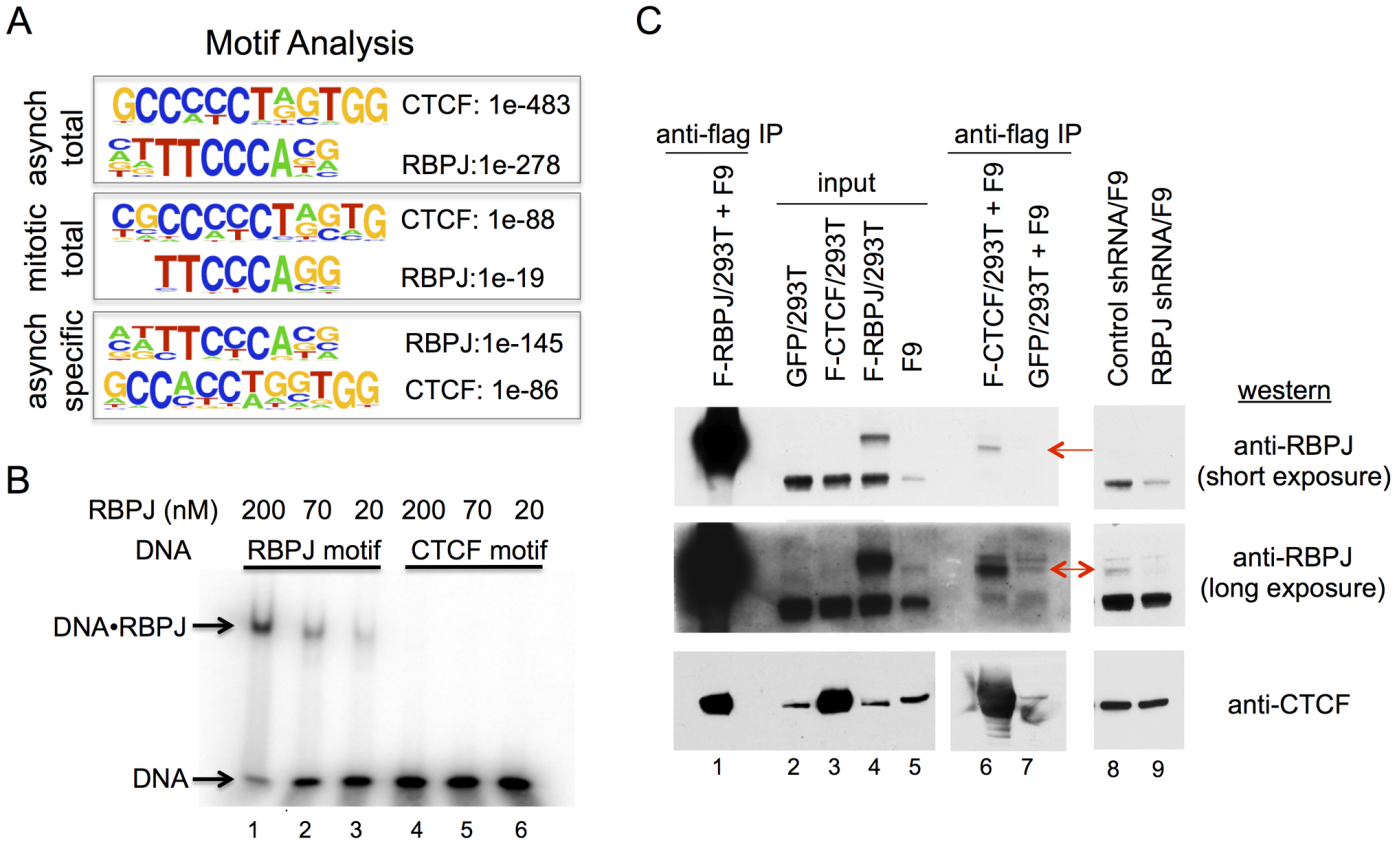
RBPJ interacts with CTCF and is enriched at genomic loci containing CTCF-binding motifs. (A) Protein-binding motifs enriched at sites of RBPJ occupancy on chromatin from asynchronous cells and mitotic cells, as well as occupancy sites unique to asynchronous cells. The enrichment p-values for each motif are as shown. No significant motif was enriched at sites of RBPJ occupancy specific to mitotic chromatin. (B) Electrophoretic mobility shift assays showing binding of RBPJ to DNA containing an RBPJ-binding motif but not to DNA containing a CTCF-binding motif. 16 bp DNA fragments were used at 1 nM and RBPJ protein concentrations are as indicated. (C) Protein interaction experiments revealing RBPJ and CTCF association. Lysates prepared from 293T cells expressing Flag-RBPJ, Flag-CTCF or GFP were incubated with anti-Flag M2 agarose beads (α-Flag). After protein binding, the beads were washed and then incubated with F9 cell lysate. Bound proteins were eluted with Laemmli buffer and resolved in a NuPAGE 7% Tris-acetate gel (lanes 1, 6 and 7). RBPJ and CTCF were detected by western blot analysis, using antibodies indicated to the right. For the CTCF western, lanes 1–5 were from a long exposure and lanes 6–7 were from a short exposure. Lanes 2 and 5 are input lysates. Lanes 8 and 9 are lysates prepared from F9 cells expressing a control shRNA or an shRNA targeting RBPJ, revealing the presence of different RBPJ isoforms in F9 cells. CTCF was used as a loading control. Arrows point to the low abundance RBPJ isoform.

Using HOMER to identify motifs, we found strong enrichment of both RBPJ- and CTCF-binding motifs in the RBPJ peaks common to both asynchronous and mitotic F9 cells (Figure 5A and Table 1). Moreover, these two motifs were found in the peaks unique to asynchronous cells; however, no significant motif enrichment was observed in the peaks unique to mitotic cells. The decrease in motif enrichment in mitotic cells may be related to the peak shifting described above. These results indicate that RBPJ not only associates with genomic regions that contain an RBPJ-binding motif, but also with regions that contain a CTCF-binding motif, and that both these sites can be occupied by RBPJ in interphase and mitotic cells (Figure 3B).

Classified GO analysis revealed that peaks containing the RBPJ-binding motif were found near genes involved in cell-fate determination pathways (Table S4B). On the other hand, peaks containing the CTCF-binding motif were found near genes involved in the immune response and cell junction regulation (Table S4C). These results raise the intriguing hypothesis that RBPJ may diversify its activity by interacting with CTCF to regulate different sets of genes associated with different biological processes. Importantly, given that RBPJ associates with mitotic chromatin, many of these genes may be marked by RBPJ for epigenetic regulation (Table S1).

### RBPJ interacts with CTCF

Our motif analysis revealed that CTCF-binding motifs were enriched at sites of RBPJ occupancy. Approximately 16% of these peaks contained a CTCF-binding motif, which is similar to the percentage of peaks that contained an RBPJ-binding motif (Table 1). CTCF is a CCCTC-binding, zinc finger protein that is involved in multiple chromatin-related functions. These functions include transcriptional activation or repression, preventing the communication between promoters and nearby enhancers or silencers by binding to insulator elements, and establishing long-range chromatin interactions [5].

The recovery of CTCF-binding motifs did not result from a non-specific interaction of the anti-RBPJ antibody with the CTCF protein, as western blot analysis revealed that the anti-RBPJ antibody did not recognize the CTCF protein, which is present in F9 cell lysates (see below). Two additional explanations might account for the enrichment of CTCF-binding motifs. First, in addition to binding to its own motif, RBPJ might also have affinity to the CTCF-binding motif. Alternatively, RBPJ may interact, either directly or indirectly, with the CTCF protein, and the recovery of DNA containing the CTCF-binding motif could be a consequence of this interaction.

To distinguish between these two possibilities, we compared the relative binding affinities of the RBPJ protein to DNA fragments containing an RBPJ-binding motif or a CTCF-binding motif identified from our ChIP-seq results. As shown in Figure 5B, RBPJ readily bound to a 16 bp DNA fragment containing an RBPJ-binding motif. On the other hand, we observed little or no binding of RBPJ to a 16 bp DNA fragment containing a CTCF-binding motif, even at very high RBPJ concentrations (200 nM). Given that peaks containing RBPJ- or CTCF-binding motifs were recovered with equal efficiency, these results argue against the possibility that RBPJ interacted with CTCF-binding elements directly.

We next determined if the recovery of DNA containing CTCF-binding motifs resulted from an interaction between RBPJ and CTCF. First, we asked if RBPJ could associate with CTCF. For these experiments, we expressed Flag-tagged CTCF or Flag-tagged RBPJ in 293T cells and then purified them using anti-Flag (M2) beads. We then incubated the immobilized proteins with F9 cell lysates, to determine if we would observe interactions with endogenous RBPJ or CTCF, respectively. As shown in Figure 5C, we observed an interaction between Flag-RBPJ and endogenous CTCF (lane 1). Reciprocally, we observed an interaction between Flag-CTCF and a low abundance RBPJ species, which may arise through alternative splicing, alternative transcription initiation or post-translational modification (lane 6). Of note, different RBPJ isoforms have been observed in F9 cells [26]. shRNA-mediated RNA interference further confirmed that this CTCF interacting protein was indeed RBPJ, as its abundance was diminished when F9 cells expressed an shRNA targeting RBPJ (lane 9) but not when cells expressed a control shRNA (lane 8). Taken together, these data support the hypothesis that RBPJ and CTCF interact. Moreover, these data suggest that this interaction may be enhanced by cell-type specific protein modifications or isoform expression.

**Figure 6:**
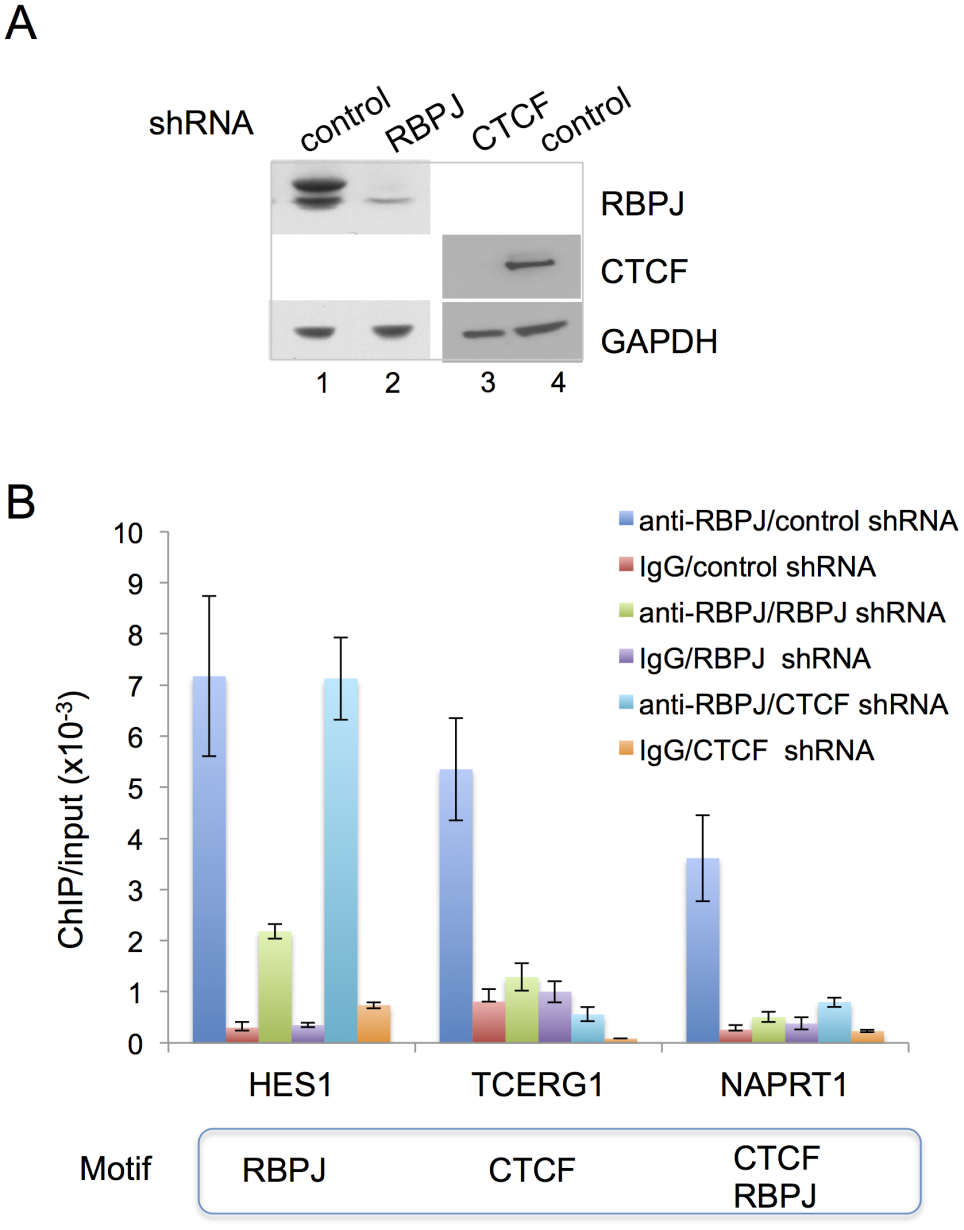
CTCF is required for the enrichment of RBPJ at the TCERG1 and NAPRT1 promoters. (A) Western blot analysis showing RNAi-mediated reduction of RBPJ or CTCF protein levels in F9 cells. F9 cells were infected with lentivirus expressing shRNA targeting RBPJ, CTCF or a control shRNA. Whole cell lysates were resolved in a NuPAGE 4–12% Bis-Tris gel (Invitrogen) and analyzed by western blot using anti-RBPJ or anti-CTCF antibodies. GAPDH was used as a loading control. (B) ChIP-qPCR analyses of F9 cells expressing RBPJ or CTCF shRNAs. F9 cells were infected with lentivirus expressing shRNA targeting CTCF, RBPJ or a control shRNA. ChIP-qPCR was used to examine RBPJ occupancy at three different loci: a region of the Hes1 promoter, which contains an RBPJ-binding motif, a region of the Tcerg1 promoter, which contains a CTCF-binding motif, and a region of the Naprt1 promoter, which contains both RBPJ- and CTCF-binding motifs (only the CTCF motif was positioned at the center of the amplicon). A region of the actin gene was used as a negative control. Primers used are described in Table S6. As expected, RBPJ RNA interference resulted in a significant decrease in signals from all three regions. CTCF RNA interference resulted in a decrease in RBPJ occupancy only at the Tcerg1 and Naprt1 regions, sites that contain CTCF-binding motifs.

Second, we used shRNA-mediated RBPJ knockdown and anti-RBPJ ChIP-qPCR to make certain that RBPJ was, indeed, interacting with the Hes1, Tcerg1 and Naprt1 promoters and that our polyclonal antibody was truly recognizing RBPJ bound to these regions. As shown in Figure 6A, we were able to substantially reduce RBPJ protein levels with shRNA targeting RBPJ. The ChIP-qPCR results shown in Figure 6B reveal that there was a substantial reduction in the signals arising from all three regions in cells expressing shRNA targeting RBPJ.

Third, we used shRNA-mediated CTCF knockdown and anti-RBPJ ChIP-qPCR to determine if CTCF was required for the association of RBPJ with DNA containing a CTCF-binding motif. As shown in Figure 6A, we were able to substantially decrease CTCF protein levels in F9 cells using shRNA-mediated RNA interference. ChIP-qPCR analysis of cells treated with a control shRNA or shRNA targeting CTCF revealed that the association of RBPJ with a region of the Hes1 promoter that contains an RBPJ-binding motif at the peak center was largely unaffected by changes in CTCF protein levels (Figure 6B). On the other hand, we observed decreased association of RBPJ with regions of the Tcerg1 and Naprt1 promoters, which contain CTCF-binding motifs at the center of the sequencing peaks (Figures 3B and S7A).

Lastly, we performed anti-CTCF ChIP-qPCR to make certain that CTCF was, indeed, bound to the Tcerg1 and Naprt1 promoters. As shown in Figure S7B, CTCF was found to occupy the Tcerg1 and Naprt1 promoters in both asynchronous and mitotic cells. Taken together, the results described above support the hypothesis that the RBPJ protein associates with CTCF-binding motifs indirectly, through an interaction with the CTCF protein.

## Discussion

Transcription factors that are retained on mitotic chromatin have the potential to function as epigenetic marks, often referred to as bookmarks, to efficiently maintain transcription programs through cell division [9], [10]. These mitotic chromatin-associated transcription factors can play important roles in the rapid reactivation of transcription or the maintenance of transcriptional repression upon exit from mitosis. In this study, we demonstrated that RBPJ, the critical transcriptional effector of the Notch signaling pathway, is retained on mitotic chromatin [11], [12]. Moreover, we found that RBPJ directly binds to nucleosomal DNA *in vitro*. The association of RBPJ with mitotic chromatin is mediated through a direct interaction with DNA, including both nucleobase and phosphodiester contacts. Interestingly, genome-wide analysis revealed that sites of RBPJ occupancy were enriched in CTCF-binding motifs, in addition to RBPJ-binding motifs, in both interphase and mitotic cells. The association of RBPJ with CTCF-binding motifs is likely mediated through the CTCF protein, as we found that RBPJ interacts with CTCF (Figure 5C) and that a decrease in CTCF expression leads to decreased RBPJ occupancy at sites harboring CTCF-binding motifs (Figure 6B). From our results, we propose that RBPJ can function as a mitotic bookmark to maintain the fidelity of transcription programs, and thus cell identity, through cell division. Additionally, by collaborating with CTCF, RBPJ may participate in establishing chromatin domains and bridging long-range chromosome interactions that can persist through mitosis.

The “default” activity of RBPJ is often considered to be transcriptional repression, and multiple mechanisms have been proposed [14], [27]. A favored hypothesis is that transcriptional corepressors or adaptor proteins bridge interactions between RBPJ and complexes containing histone deacetylases (HDACs), which would lead to the removal of acetyl groups from histones to generate repressed chromatin states. A second mechanism involves the interaction between RBPJ and the histone demethylase KDM5A, which would lead to a decrease in histone H3 lysine 4 methylation and the establishment of repressed chromatin [28]. A third mechanism involves the cooperation of RBPJ and a polycomb repressive complex [29], which would lead to chromatin compaction at sites of RBPJ occupancy.

During transcriptional activation, the RBPJ-associated proteins that direct repression are exchanged for proteins that mediate transcriptional activation. The components of the core activation complex can vary, but it always contains RBPJ, a Mastermind-like protein (MAML) and the intracellular domain of a Notch receptor (NICD) [14], [27], [30]. NICD arises from the release of the intracellular domain of the Notch receptor from the cell surface upon receptor-ligand engagement [12], [15]. Although the actual mechanism that leads to transcriptional activation is still unclear, the recruitment of histone acetyl transferases, such as KAT2A, KAT2B and p300, by the core complex is an important component of the activation process [31]–[33].

We do not yet know if RBPJ functions in F9 cells solely as a transcriptional repressor, solely as a transcriptional activator or as both a repressor and activator. In the classic pathway, activation is achieved when a Notch receptor, expressed on the surface of one cell, engages with a ligand, expressed on the surface of an adjacent cell. Several Notch receptors and Notch ligands are expressed in F9 cells ([34], Lake and Fan unpublished observations) and, therefore, Notch signaling could conceivably be activated to some degree. Importantly, several Notch regulated genes were identified as sites of RBPJ occupancy in F9 cells (Figure S8). Future experiments aimed at determining the impact of mitosis-specific knockdown of RBPJ protein levels on the faithful propagation of transcription programs through cell division will reveal the function of RBPJ in the maintenance of transcription memory regulated by Notch signaling.

Our genome-wide studies in the murine embryonal carcinoma cell line F9 revealed that approximately 60% of the RBPJ occupancy sites in cycling cells are maintained on mitotic chromatin, and this accounts for approximately 80% of the total RBPJ occupancy sites observed in mitotic cells. The other 40% of RBPJ occupancy sites that are unique to interphase chromatin (Figure 3A) might represent a class of genes whose regulation is maintained in a more dynamic state, as had been observed in Drosophila cells [35]. These more dynamic interactions perhaps provide plasticity to RBPJ-mediated transcription regulation and, thus, cell-fate determination. The function, if any, of RBPJ occupancy at sites that are unique to mitotic cells, which represents approximately 20% of mitotic chromatin-RBPJ associations, waits to be determined.

Gene annotation revealed that RBPJ occupies both intergenic and genic regions in both mitotic and asynchronous F9 cells (Figure S6C), and that the relative distribution of occupancy between these classes of genetic elements does not substantially change through the cell cycle. Our results also revealed that most RBPJ occupancy sites lie in intergenic and intronic regions, and these results are similar to those obtained with the Epstein-Barr virus immortalized murine lymphoblastic cell line IB4 [36]. Interestingly, a study of RBPJ occupancy in the human T-lymphoblastic leukemia cell line CUTLL1, which has an activating Notch1 mutation, revealed that most RBPJ occupancy sites lay in promoter regions [37]. This difference in the genomic distribution of RBPJ occupancy sites may result from the aberrant Notch1 activation that occurs in CUTTL1 cells, or it may simply reflect the impact of cellular context on Notch/RBPJ-dependent processes [16].

To examine the variability of RBPJ occupancy between different cell lines, we have directly compared our data obtained with the embryonal carcinoma cell line F9, to the published data sets obtained with the murine TLL cell lines, T6E and G4A2 (Figure S9A) [37]. From the TLL study alone, only ∼15% of the RBPJ occupancy sites were common between the T6E and G4A2 cell lines. We found that ∼7% of the RBPJ occupancy sites in F9 cells are common to either of the TLL cell lines. Therefore, from these study comparisons, it can be seen that the bulk of RBPJ occupancy can vary widely between cell lines, and RBPJ is not exclusively restricted to sites containing an RBPJ-binding motif.

Examples of common RBPJ occupancy sites at five genomic loci in the three cell lines (F9, T6E and G4A2) are shown in Figure S9B. Timm 44 is an example of a common interphase-specific RBPJ occupancy site and the others are examples of sites occupied by RBPJ in both interphase and mitosis. Additionally, it can be seen that RBPJ can occupy a region of the Tcerg1 and Gipc1 genes in both F9 and T6E cells, a region that contains only a CTCF-binding motif and not an RBPJ-binding motif. This latter observation supports the notion that RBPJ and CTCF might have an overlapping function.

As described above, evidence from our study and the studies of others reveals that RBPJ does not bind only to genomic regions that contain an RBPJ-binding motif. And, it is clear that RBPJ does not occupy all potential RBPJ-binding sites. The interaction of RBPJ with the Naprt1 promoter is particularly interesting, as this region contains both RBPJ- and CTCF-binding motifs. Our observation that CTCF knockdown decreases RBPJ occupancy at the Naprt1 locus indicates that the RBPJ-binding motif is not playing the prominent role at this region. Instead, it appears that RBPJ associates with the Naprt1 locus indirectly through an interaction with the CTCF protein. The importance of the CTCF-binding motif in directing RBPJ occupancy at the Tcerg1 promoter is further supported by the fact that the CTCF motif lies in the center of the sequencing peak (Figure S7A).

Similarly, it is not clear why RBPJ occupies only specific CTCF sites, given that there are over 50,000 sites that are occupied by CTCF in any given cell [38]. CTCF is often considered a multivalent protein, as it contains 11 zinc fingers. It is possible that the binding of CTCF through a particular combination of zinc-fingers may play a role in directing RBPJ-CTCF associations at specific loci, possibly through regulating the exposure of an interaction surface. Other possible mechanisms that might direct the association of RBPJ with specific CTCF-binding motifs could be post-translational modifications of RBPJ or CTCF, local chromatin structure at the binding sites, or long-range chromatin organization. Currently, the mechanisms that control RBPJ target-site selection are essentially unknown, but it is becoming increasingly apparent from genome-wide studies that, in addition to the underlying DNA sequence, other factors will likely play instrumental roles in stabilizing RBPJ-chromatin associations.

Although RBPJ occupancy within the vicinity of genes is likely to be important for transcription regulation, the role of RBPJ at intergenic regions is much more enigmatic. These intergenic regions may function as distal enhancer elements. Furthermore, given the interaction between RBPJ and CTCF that we observed (see below), it is possible that the association of RBPJ with intergenic regions may also play a role in organizing long-range chromatin structure. As expected from the known functions of Notch signaling, gene ontology indicates that RBPJ is likely to function in stem cell maintenance, development, cell differentiation, metabolism and the processing of non-coding RNAs.

Our *in vitro* nucleosome binding studies revealed that RBPJ preferentially binds close to the DNA entry/exit sites of a core nucleosome, as opposed to sites that lie at the DNA center. The observed preference of RBPJ binding might be due to the relatively weaker DNA-histone contacts in this region [39], [40], and thus DNA located at the nucleosome ends would be more readily exposed through “breathing” than nucleosomal DNA located near the dyad. However, it is also possible that the rotational phasing of an RBPJ-binding motif on the nucleosome surface might also play a role in determining the binding-site preference [41]. Importantly, the binding of RBPJ close to the entry/exit sites of a nucleosome could initiate the binding of other transcription co-activators or co-repressors to more internal sites upon mitotic exit through cooperative interactions [42]. Moreover, the binding of RBPJ to nucleosomes may be instrumental to the targeting of histone modifying or nucleosome remodeling enzymes to specific nucleosome for efficient transcriptional activation or repression. It is also formally possible that the preferential binding of RBPJ to the entry/exit sites of nucleosomes may help position nucleosomes around RBPJ occupancy sites. Additional *in vitro* and *in vivo* experiments are needed to test these hypotheses.

We do not yet know the cause of the shift in RBPJ occupancy sites that occurs in mitotic cells, but it is most likely related to mitotic chromatin condensation. Such shifting could be the consequence of nucleosome repositioning that might be needed to reorganize chromatin structure to permit chromatin compaction or to silence transcription. For instance, a shifting of H2AZ-conatining nucleosomes had been observed to occur during mitosis. In this case, H2AZ nucleosomes at the +1 position were observed to migrate to the transcription start site, and it was hypothesized that this shifting was important to occlude the start site from the transcriptional machinery to shut down transcription. Additionally, DNase I hypersensitive sites have been observed to shift position between interphase and mitotic chromatin [43]. Regardless of the underlying mechanism that causes the shifting of RBPJ occupancy during mitosis, by maintaining its chromatin association, RBPJ would be able to find its preferred occupancy sites more efficiently upon mitotic exit by sliding along DNA; such a mechanism would reduce the complexity of target-site search from three dimensions to one.

The interaction that we observed between RBPJ and CTCF was readily apparent when immobilized RBPJ was used to capture endogenous CTCF from an F9 cell lysate. When immobilized CTCF was used to capture endogenous RBPJ, the interaction was restricted to a low abundance RBPJ species. This observation suggests that the RBPJ-CTCF interaction may be enhanced through cell-type specific RBPJ isoform expression or post-translational modification. It is also possible that the RBPJ-CTCF interaction might be enhanced by the post-translational modification of CTCF, and if such a modification occurred at a low level, much of the exogenously expressed CTCF protein would be unmodified and, therefore, unable to support an interaction with RBPJ. Additionally, we cannot exclude the possibility that the interaction of RBPJ with CTCF leads to epitope occlusion, thus decreasing the efficiency of complex purification through immunoprecipitation of the CTCF protein.

Our findings that CTCF-binding motifs are enriched at sites of RBPJ occupancy on both interphase and mitotic chromatin, and that RBPJ and CTCF interact, raise the intriguing hypothesis that RBPJ and CTCF may coordinate their activities to regulate gene expression. The association between RBPJ and CTCF activities may be limited to specific cellular contexts, as CTCF-binding motifs were not enriched at sites of RBPJ occupancy in cells of the lymphoid lineage [36], [37]. Reciprocally, the non-RBPJ-binding motifs that were enriched in lymphoid cells were not enriched in F9 cells. It is possible that a functional relationship between RBPJ and CTCF is restricted to developmentally early cells, represented by the F9 embryonal carcinoma cell line, in which both CTCF and RBPJ play prominent roles. Taken together, these observations raise the intriguing possibility that by interacting with different transcription factors in different cellular contexts, the activities of RBPJ, and hence Notch signaling, could be greatly diversified through differential chromatin targeting.

Like RBPJ, CTCF is retained on mitotic chromatin [44], and long-range chromatin interactions mediated by CTCF appear to be maintained in mitosis [6]. Given that the association of RBPJ with CTCF-binding motifs is likely to be mediated by the CTCF protein, we propose that RBPJ can collaborate with CTCF to bridge long-range chromatin interactions that can be propagated through cell division to regulate gene expression and maintain cell identity. Future studies using chromosome conformation capture techniques [45] will be critical to determine the functions of the RBPJ-CTCF interaction in chromatin structure regulation and gene expression during both interphase and mitosis.

## Materials and Methods

### Construct generation, protein purification and antibody production

Constructs encoding GFP-, GST- and Flag-tagged mouse RBPJ were generated by PCR amplification and cloned into pLenti-PGK [46] or pMSCV (Clontech) for expression in mammalian cells, and in pFastBac1 for expression insect cells. The cDNA encoding the mouse RBPJ isoform 2 (NP_001074396.1) was obtained from Thermo Scientific. For protein expression in SF9 cells, Flag-tagged RBPJ was purified using M2-affinity chromatography [47]. RBPJ mutations were generated with the QuikChange Site-Directed Mutagenesis Kit (Agilent).

For generating anti-mRBPJ antibodies, GST-mRBPJ was expressed in SF9 cells and purified using glutathione sepharose chromatography. The rabbit anti-RBPJ polyclonal antibody was generated by Cocalico Biologicals, Inc. Characterization of the anti-RBPJ antibody is shown in Figure S4.

### Live F9 cell imaging

F9 cells were transiently transfected with eGFP-RBPJ or its derivatives using lipofactamine 2000 (Invitrogen). Twenty hours post-transfection, mitotic cells were enriched by mitotic cell shake-off. Live cells were counterstained with Hoechst 33342 (Invitrogen) and images were collected using a Leica DM6000B microscope, equipped with DAPI ET, k (material number 11504203) and L5 ET, k (material number 11504166) filter cubes. Hoechst 33342 was imaged using BP 404/20 and BP 457/20 excitation and suppression filters, respectively. eGFP was imaged using BP 480/40 and BP 527/30 excitation and suppression filters, respectively.

To quantify the fraction of RBPJ and its derivatives associated with mitotic chromatin, the eGFP intensity was measured using Image J software [48]. eGFP signals associated with mitotic chromatin were identified by their overlap with Hoechst 33342. For each cell, signal intensities were measured from five different regions on mitotic chromatin, in the nucleocytoplasm, and outside the cell (background). Average intensities for each region were calculated, and average eGFP intensities for mitotic chromatin and the nucleocytoplasm were adjusted by subtracting average background intensity. The ratio of mitotic chromatin-associated eGFP intensity versus nucleocytoplasm-associated eGFP intensity was then determined.

### Nucleosome assembly

Three 147 bp DNA fragments containing two 20-bp GT phasing sequences, located at position 1–40 (Figure 2A) [49], were assembled into mononucleosomes with HeLa cell histones, using step-gradient salt dialysis [50]. The RBPJ-end and RBPJ-dyad DNA fragments contain the RBPJ-binding motif (TTCCCACA) at positions 127–134 (entry/exit) and 57–64 (dyad), respectively. The RBPJ-minus fragment contains no RBPJ-binding motif. DNA fragments used for assembly were generated by PCR and body-labeled with [^32^P] α-dATP.

### Electrophoretic mobility shift assays (EMSA)

Proteins were mixed with DNA or mononucleosomes at the indicated concentrations. Binding reactions were carried out in 12 mM Hepes (pH 7.9), 10 mM Tris⋅HCl (pH 7.5), 60 mM KCl, 8% glycerol, 4 mM MgCl_2_, and 0.02% NP40 at 30°C. Reactions were loaded directly onto a 5% polyacrylamide gel-0.5× TBE. Competitor DNA was used as noted in the Figures.

For gel shifts described in Figure 5B, 16 bp DNA fragments were generated by annealing two complementary DNA oligonucleotides. The annealed DNA fragments were labeled with ^32^P using T4 polynucleotide kinase. The sequence of the DNA fragments containing the RBPJ and CTCF motif were CACTGGGAACCTACCC and GGCCACTAGGGGGCGC, respectively.

### Cell culture and cell synchronization

F9 cells were cultured in DMEM medium supplemented with 10% FBS. To enrich for mitotic cells, F9 cells were treated with 1 µg/ml nocodazole (Sigma) for 4 hours. Mitotic cells were collected by gently washing the loosely adherent mitotic cells off the culture dishes with PBS. After fixation, cells were stained with DAPI to determine the fraction of mitotic cells. In general, for the chromatin immunoprecipitation experiments, the mitotic index was greater than 98% (see Figure S5).

### shRNA knockdown

Mission non-targeting shRNA controls (SHC002), shRNA targeting RBPJ (TRCN0000097288, Sigma) or shRNA targeting CTCF (kindly provided by Marisa Bartolomei, U. Penn) were co-transfected with third generation lentivirus packaging plasmids into 293T cells. The culture medium was changed 24 hours post-transfection, and virus was collected 24 hours later. Infected F9 cells were harvested 48 to 60 hours post-infection.

### Co-IP, ChIP and real-time PCR experiments

pCDNA3 constructs expressing Flag-tagged CTCF or Flag-tagged RBPJ were transfected into 293T cells. Forty-eight hours post transfection cells were lysed in PBS/0.05% Triton X-100/protease inhibitors and sonicated. After clarification of the soluble fraction by centrifugation, lysates were incubated overnight with anti-Flag (M2) agarose (Sigma) at 4°C. The beads were then washed four times with PBS/0.05% Triton X-100 and immobilized Flag-tagged CTCF or Flag-tagged RBPJ were mixed with F9 cell lysates, prepared as described above, and incubated overnight. After four washes, protein complexes were eluted with Laemmli buffer and resolved on a 7% Tris-acetate gel (Invitrogen). Western blots were probed with a rabbit anti-CTCF antibody (Millipore Cat#07-729) or the rabbit anti-RBPJ antibody. 293T cells were used as an expression system for the Flag-tagged proteins, as F9 cells transfect with a much lower relative efficiency.

Chromatin immunoprecipitations were carried out as previously described [47]. In brief, chromatin was sonicated to achieve a DNA fragment length of ∼200–500 bps. Chromatin equivalent to 70 µg of DNA was incubated with 100 µg of rabbit IgG (Sigma) or 10 µl of rabbit anti-RBPJ antibody (which we estimated to contain ∼100 µg total IgG). ChIPed DNA was analyzed by real-time PCR using a Bio-Rad MyiQ system and SYBR green. Primers were as described in Table S6.

### ChIP-Seq and data analysis

10 ng of ChIPed DNA was used to prepare libraries for deep sequencing, using the multiplexed ChIP-Seq sample preparation protocol described on the website of the Next-Generation Sequencing Core, Perelman School of Medicine, University of Pennsylvania (http://ngsc.med.upenn.edu/). The Next-Generation Sequencing Core at University of Pennsylvania performed DNA sequencing. For each cell population (asynchronous and mitotic), two ChIP-seq replicates and one input control were analyzed: there were a total of six sequencing data sets. Raw and processed files (GSE45889) have been deposited at the Gene Expression Omnibus (GEO) repository (http://www.ncbi.nlm.nih.gov/geo/query/acc.cgi?token=lxchbcosuiwwsvw&acc=GSE45889).

ChIP-seq reads were mapped to the mouse genome (mm9) using bowtie with options “--best -v 2 --strata –m 1” to obtain unique alignments with up to two mismatches [22]. We obtained ∼35 million mapped reads for the anti-RBPJ ChIP samples. Peak calling was carried out using HOMER (Hypergeometric Optimization of Motif EnRichment) with a default option (FDR = 0.001 and Poisson p-value cutoff = 0.0001) on ChIPed samples against the matching input samples, and then a 1 RPM cutoff was applied [23].

To identify RBPJ binding sites specific to asynchronous or mitotic cells, we used mitotic data or asynchronous data as background, respectively, and we used the “getDifferentialPeaks –size 200” command of HOMER. A specific peak was defined as having an enrichment within a 200 bp region of more than 4-fold between two cell populations and a Poisson enrichment p-value<0.0001. The remaining peaks were defined as common.

To generate the histogram in Figure 4D, we calculated the distance of all asynchronous peaks to their closest mitotic peak. We counted the number of peaks every 50 bps.

RBPJ occupancy sites were annotated with the following priority: (1) promoter (from −1 kb to +100 bp around the transcription start site), (2) TTS (from −100 bp to +1 kb around the transcription termination site), (3) Exon, (4) Intron, (5) dTSS (distal promoter, from −10 kb to +1 kb around TSS) and (6) Intergenic (all RBPJ occupancy sites that did not fall into categories 1–5) (Figure S6 and Tables S2, S3) [23].

## Supporting Information

Figure S1 Quantification of signal intensities from mitotic chromatin bound versus unbound RBPJ and RBPJ derivatives shown in Figure 1. Dots represent ratios from individual cells (bound/unbound). Horizontal bars represent mean values for each data set. Mean values plus SEM are as follows: 3.6+/−0.2 (RBPJ), 1.6+/−0.04 (K153A, S182A), 1.3+/−0.04 (R179A, R181A), and 1.1+/−0.03 (R52A, K153A, S182A).
(TIFF)

Figure S2 RBPJ preferentially binds to DNA containing an RBPJ-binding motif. (A) Two naked DNA fragments of 147 bp were used in the gel-shift assays: one contains an RBPJ-binding motif at position 127–134 and the other does not contain an RBPJ-binding motif. (B) Flow chart of experimental scheme. (C) Reactions were resolved in a 5% native polyacrylamide gel. The 147 bp DNA fragment that does not contain the RBPJ-binding motif was used as unlabeled DNA competitor.
(TIFF)

Figure S3 Titration of RBPJ for nucleosome binding assays. Two core mononucleosomes were used in the binding assays: RBPJ-end contains an RBPJ-binding motif at positions 127–134, which lies close to the entry/exit sites of the nucleosomal DNA, and RBPJ-minus nucleosomes, which do not contain an RBPJ-binding motif. Varying amounts of RBPJ were used in the binding assays as indicated.
(TIFF)

Figure S4 Characterization of the rabbit anti-RBPJ antibody. (A) Anti-RBPJ antibody specificity as revealed by western blot analysis. F9 cells treated with shRNA targeting RBPJ (+) or a non-specific shRNA (−) for 60 hours. Lysates were resolved in a NuPAGE 4–12% Bis-Tris gel, and western blots were probed with the anti-RBPJ and an anti-GAPDH antibody. (B) Western blot analysis showing RBPJ immunoprecipitation from crosslinked cells. 293T cells expressing Flag-RBPJ were cross-linked with 1% formaldehyde, and after sonication lysates were subjected to immunoprecipitation with the rabbit anti-RBPJ antibody or control rabbit IgG. The input to IP ratio loaded on the gel was 1∶4. The western blot was probed with a mouse anti-Flag antibody (M2).
(TIFF)

Figure S5 Purity of mitotic cell preparations as revealed by immunofluorescence microscopy. Nocodazole arrested F9 cells were immunostained with antibodies against serine 10 phosphorylated histone H3 (green) and elongating RNA polymerase II (red). DNA was counterstained with DAPI. The field shown contains about 85 mitotic cells and no interphase cells, indicating that this mitotic cell preparation was greater than 98% pure.
(TIFF)

Figure S6 Pie charts illustrating the genomic distribution of RBPJ occupancy, as determined by gene annotation. (A) Distribution of total RBPJ occupancy on chromatin of asynchronous cells. (B) Distribution of total RBPJ occupancy on mitotic chromatin. (C) Distribution of RBPJ occupancy common to asynchronous and mitotic cells. (D) Distribution of RBPJ occupancy unique to asynchronous cells. (E) Distribution of RBPJ occupancy unique to mitotic cells.
(TIFF)

Figure S7 Association of the CTCF protein with the Naprt1 and Tcerg1 promoters, as revealed by ChIP-qPCR. (A) Naprt1 contains both CTCF- and RBPJ-binding motifs (shown in red and blue, respectively), with the CTCF-binding motif positioned at the center of the RBPJ ChIP sequencing peak. (B) Anti-CTCF ChIP-qPCR demonstrating that CTCF binds to the Naprt1 and Tcerg1 promoters, but not to Hes1 or actin, in both asynchronous and mitotic F9 cells.
(TIFF)

Figure S8 RBPJ binds to Notch responsive genes in asynchronous and mitotic F9 cells. Screen shots from the UCSC Genome Browser revealing RBPJ occupancy at Notch responsive genes. The position of the RBPJ-binding motif within each peak is indicated with an asterisk. The transcription factors Hes7 (A) and HeyL (B) are representative Notch-target genes of the Hes and Hey families. Timm13 (C), Nmnat2 (D) and Fbxl19 (E) are from Castel and Mourikis et al. [51]. Coordinates of the regions shown are (A) chr11: 68,930,148-68,935,117, (B) chr4:122,908,000-122,912,999, (C) chr10:80,359,119-80,366,062, (D) chr1:154,936,858-154,940,532, and (E) chr7:134,889,218-134,893,011.
(TIFF)

Figure S9 Comparisons of RBPJ ChIP-seq results obtained from F9 cells to those of the TLL cell lines, T6E and G4A2. (A) Venn diagram illustrating the overlap of RBPJ occupancy sites in F9 cells (this study) and in T6E and G4A2 cells [37]. (B) Screen shots taken from the UCSF Genome Browser showing side-by-side comparison of RBPJ occupancy at five regions. Also included are duplicates from asynchronous and mitotic F9 cells as well as input controls. The RBPJ- and CTCF-binding motifs are marked with black and pink squares, respectively. The coordinates of these five loci from left to right are (1) chr16:30,055,655-30,076,174, (2) chr14:76,549,884-76,555,073, (3) chr8:86,183,791-86,188,980, (4) chr8:4,273,375-4,278,564 and (5) chr18:42,668,310-42,675,184. Region 1, 4 and 5 contain the promoters of Hes1, Timm44 and Tcerg1, respectively. No transcript is found associated with region 2, and the peaks shown in region 3 lay in an intron of Gipc1.
(TIFF)

Table S1 GO analysis of RBPJ binding peaks common to asynchronous and mitotic F9 cells.
(XLSX)

Table S2 Annotation of RBPJ binding peaks in asynchronous F9 cells.
(XLSX)

Table S3 Annotation of RBPJ binding peaks in mitotic F9 cells.
(XLSX)

Table S4 GO analysis of RBPJ binding peaks in asynchronous F9 cells. (A) All RBPJ peaks. (B) RBPJ peaks containing the RBPJ binding motif. (C) RBPJ peaks containing the CTCF binding motif. (D) RBPJ peaks containing RBPJ and CTCF binding motifs.
(XLSX)

Table S5 GO analysis of RBPJ binding peaks in mitotic F9 cells. (A) All RBPJ peaks. (B) RBPJ peaks containing RBPJ and CTCF binding motifs. (C) RBPJ peaks containing the CTCF binding motif. (D) RBPJ peaks containing the RBPJ binding motif.
(XLSX)

Table S6 Primers used in real-time PCR.
(DOCX)
