# Correction: RBPJ, the Major Transcriptional Effector of Notch Signaling, Remains Associated with Chromatin throughout Mitosis, Suggesting a Role in Mitotic Bookmarking

**DOI:** 10.1371/journal.pgen.1006209

**Published:** 2016-07-18

**Authors:** The PLOS Genetics Staff

In the PDF of this manuscript, the footnote to accompany the yin-yang symbol next to the first two authors’ names is omitted. The first two authors, Robert J. Lake and Pei-Feng Tsai, should be noted as contributing equally to this work. The publisher apologizes for the error.
