# Supplementary figures and images for: RBPJ, the Major Transcriptional Effector of Notch Signaling, Remains Associated with Chromatin throughout Mitosis, Suggesting a Role in Mitotic Bookmarking
Source: PLoS Genet. 2014 Mar 6;10(3):e1004204. doi: 10.1371/journal.pgen.1004204 (PMC3945225; doi:10.1371/journal.pgen.1004204)

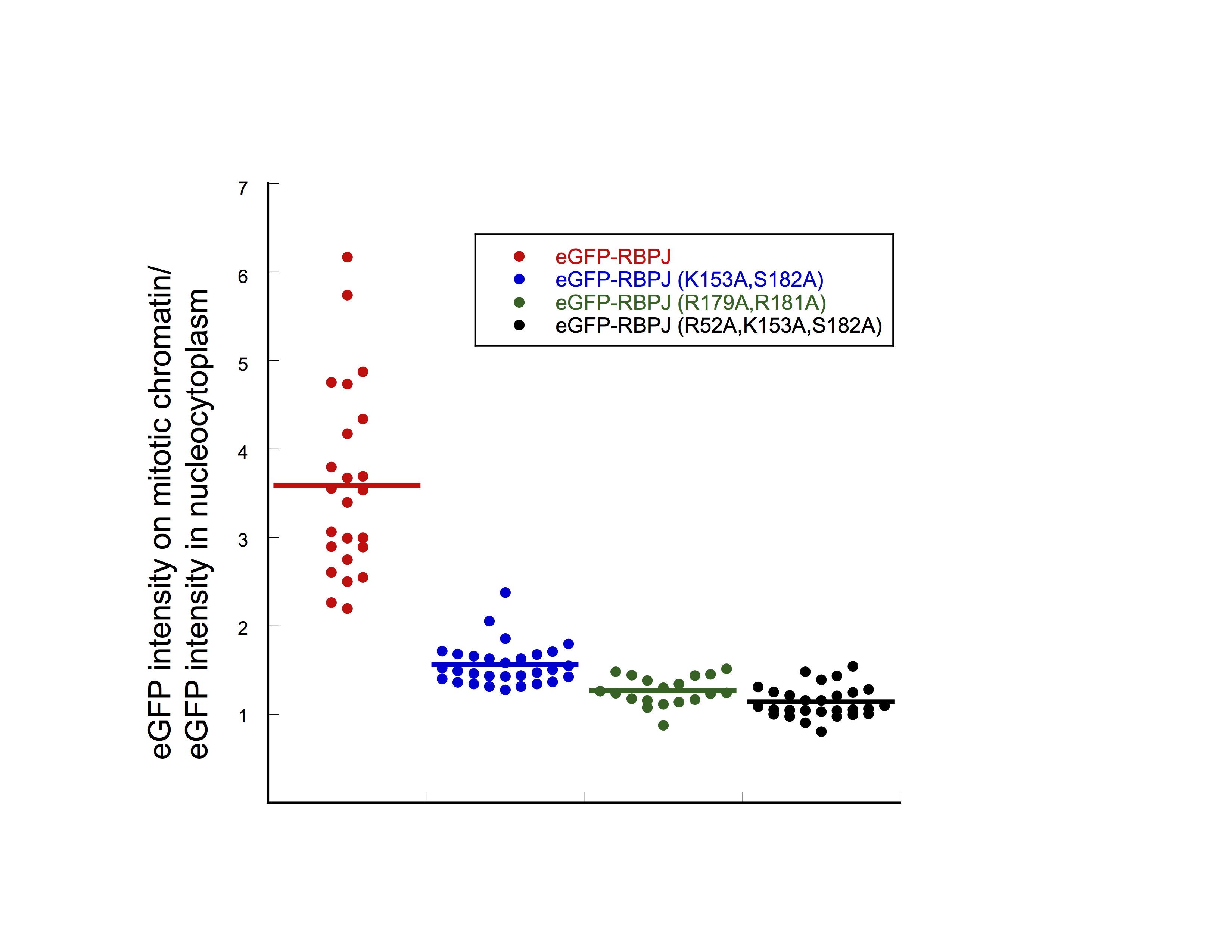

Supplement: Figure S1 — Quantification of signal intensities from mitotic chromatin bound versus unbound RBPJ and RBPJ derivatives shown in Figure 1. Dots represent ratios from individual cells (bound/unbound). Horizontal bars represent mean values for each data set. Mean values plus SEM are as follows: 3.6+/−0.2 (RBPJ), 1.6+/−0.04 (K153A, S182A), 1.3+/−0.04 (R179A, R181A), and 1.1+/−0.03 (R52A, K153A, S182A). (TIFF) [file pgen.1004204.s001.tif]

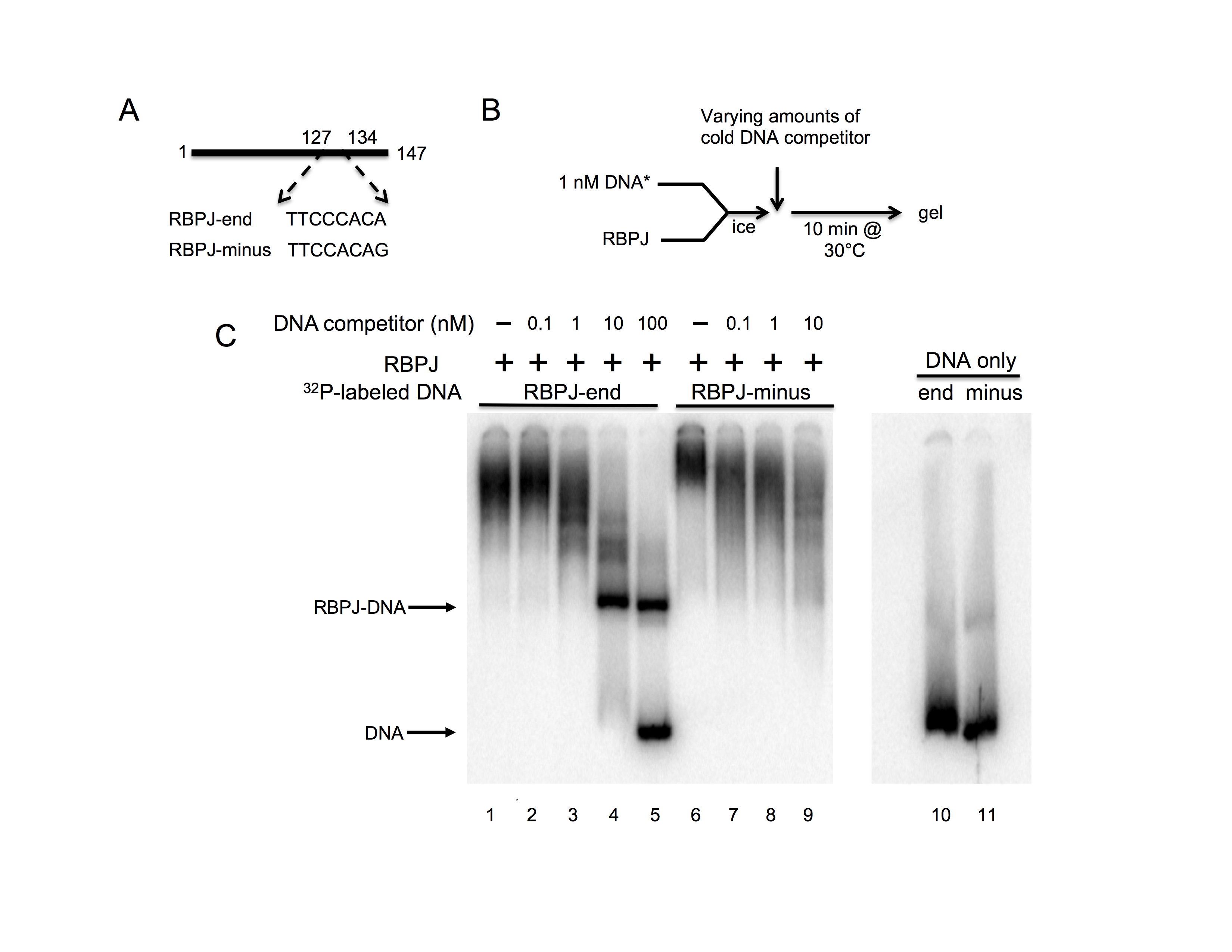

Supplement: Figure S2 — RBPJ preferentially binds to DNA containing an RBPJ-binding motif. (A) Two naked DNA fragments of 147 bp were used in the gel-shift assays: one contains an RBPJ-binding motif at position 127–134 and the other does not contain an RBPJ-binding motif. (B) Flow chart of experimental scheme. (C) Reactions were resolved in a 5% native polyacrylamide gel. The 147 bp DNA fragment that does not contain the RBPJ-binding motif was used as unlabeled DNA competitor. (TIFF) [file pgen.1004204.s002.tif]

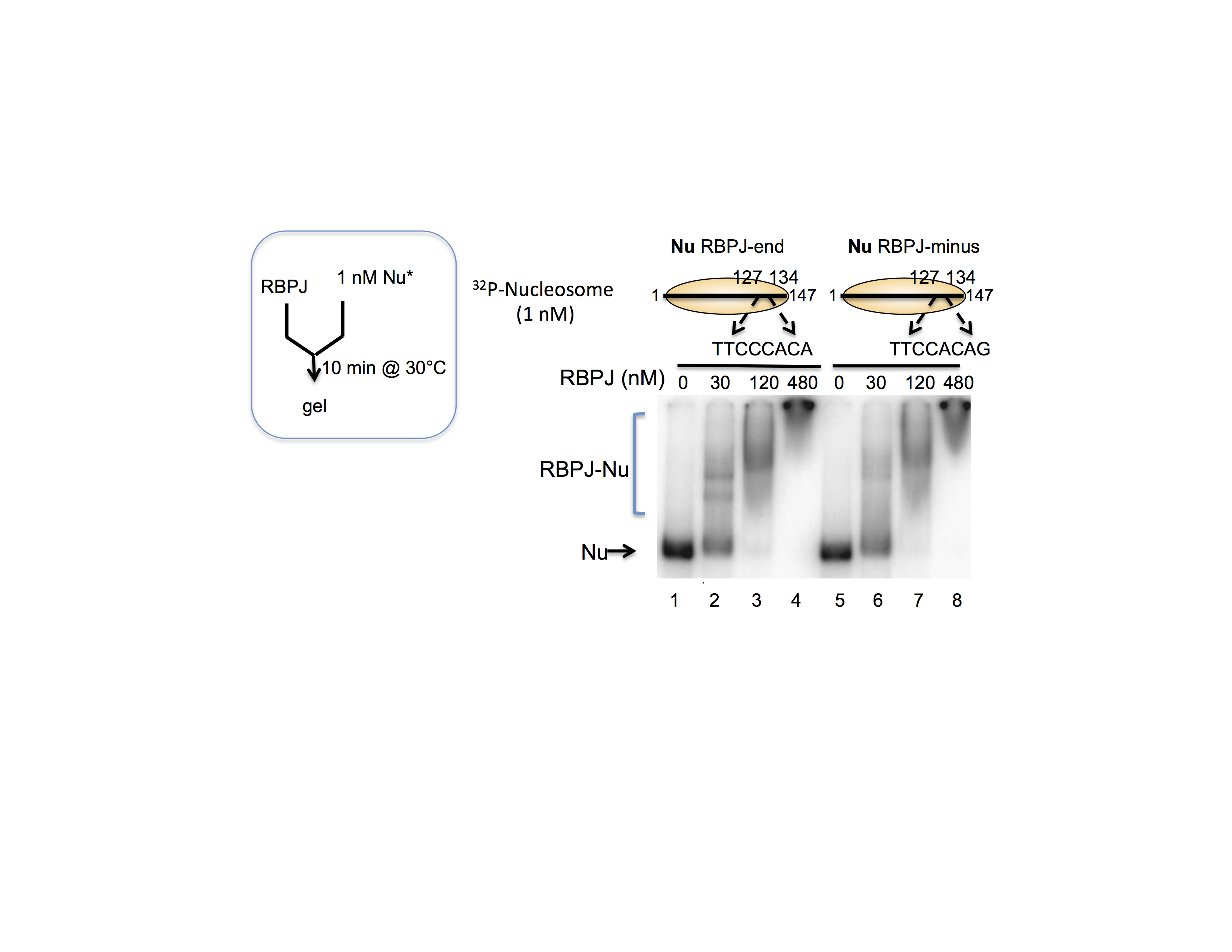

Supplement: Figure S3 — Titration of RBPJ for nucleosome binding assays. Two core mononucleosomes were used in the binding assays: RBPJ-end contains an RBPJ-binding motif at positions 127–134, which lies close to the entry/exit sites of the nucleosomal DNA, and RBPJ-minus nucleosomes, which do not contain an RBPJ-binding motif. Varying amounts of RBPJ were used in the binding assays as indicated. (TIFF) [file pgen.1004204.s003.tif]

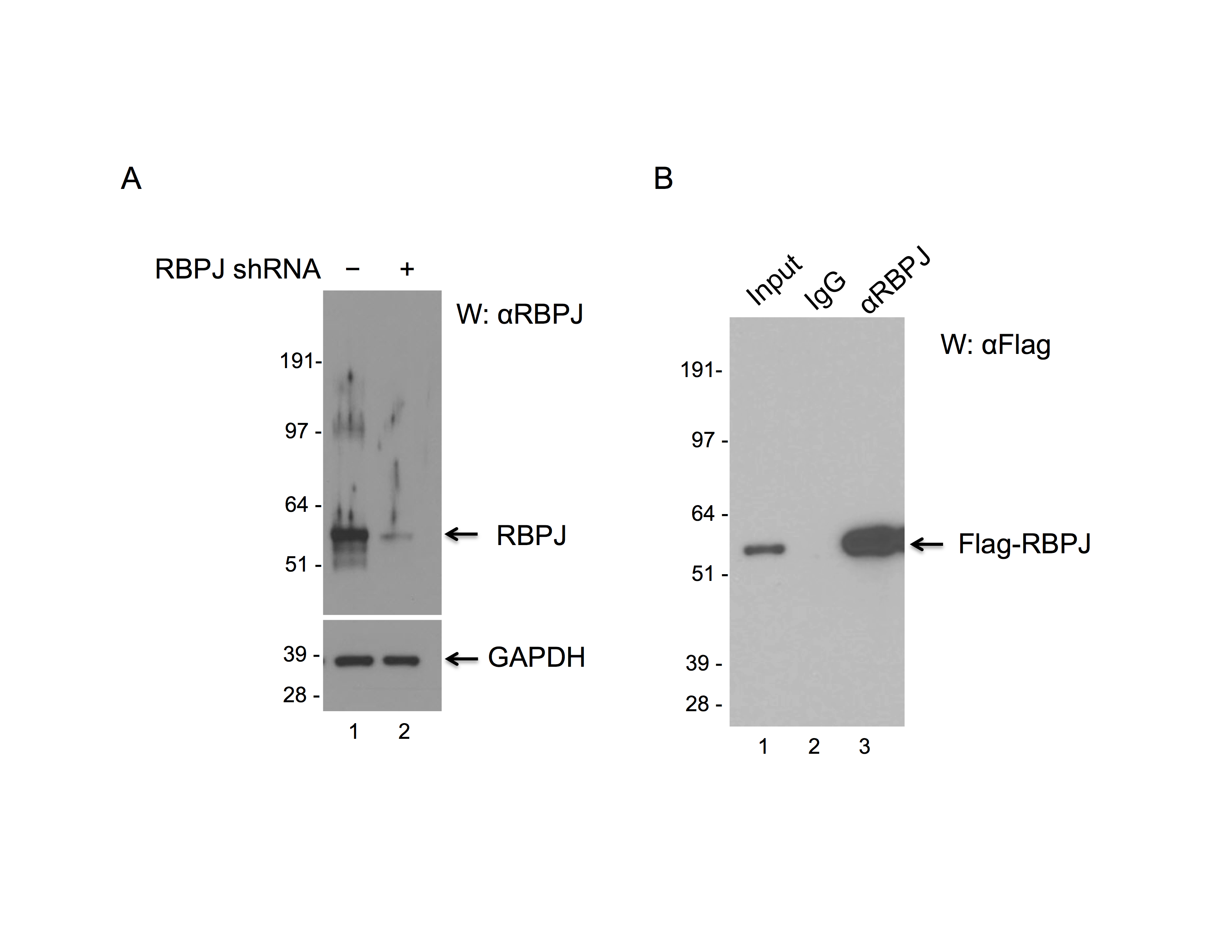

Supplement: Figure S4 — Characterization of the rabbit anti-RBPJ antibody. (A) Anti-RBPJ antibody specificity as revealed by western blot analysis. F9 cells treated with shRNA targeting RBPJ (+) or a non-specific shRNA (−) for 60 hours. Lysates were resolved in a NuPAGE 4–12% Bis-Tris gel, and western blots were probed with the anti-RBPJ and an anti-GAPDH antibody. (B) Western blot analysis showing RBPJ immunoprecipitation from crosslinked cells. 293T cells expressing Flag-RBPJ were cross-linked with 1% formaldehyde, and after sonication lysates were subjected to immunoprecipitation with the rabbit anti-RBPJ antibody or control rabbit IgG. The input to IP ratio loaded on the gel was 1∶4. The western blot was probed with a mouse anti-Flag antibody (M2). (TIFF) [file pgen.1004204.s004.tif]

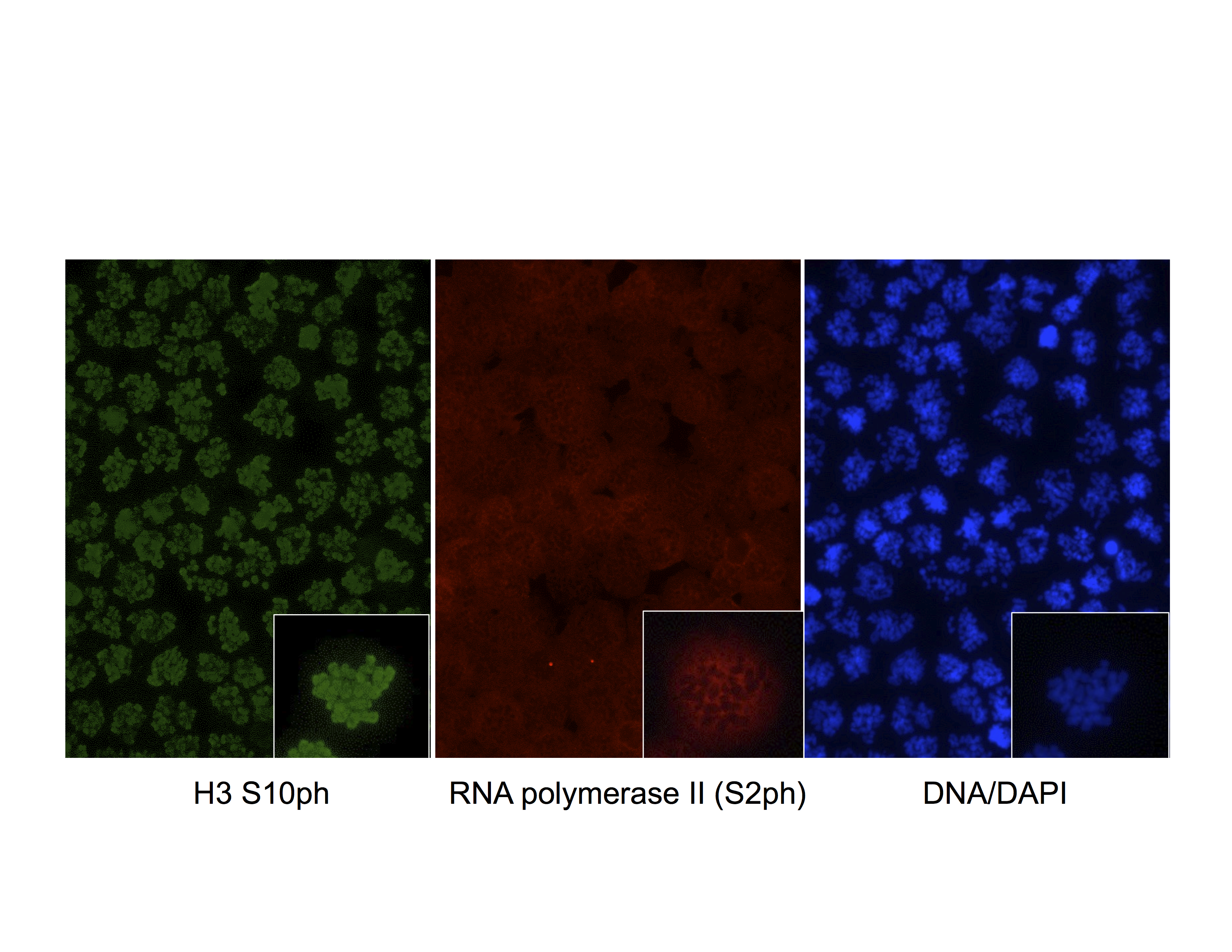

Supplement: Figure S5 — Purity of mitotic cell preparations as revealed by immunofluorescence microscopy. Nocodazole arrested F9 cells were immunostained with antibodies against serine 10 phosphorylated histone H3 (green) and elongating RNA polymerase II (red). DNA was counterstained with DAPI. The field shown contains about 85 mitotic cells and no interphase cells, indicating that this mitotic cell preparation was greater than 98% pure. (TIFF) [file pgen.1004204.s005.tif]

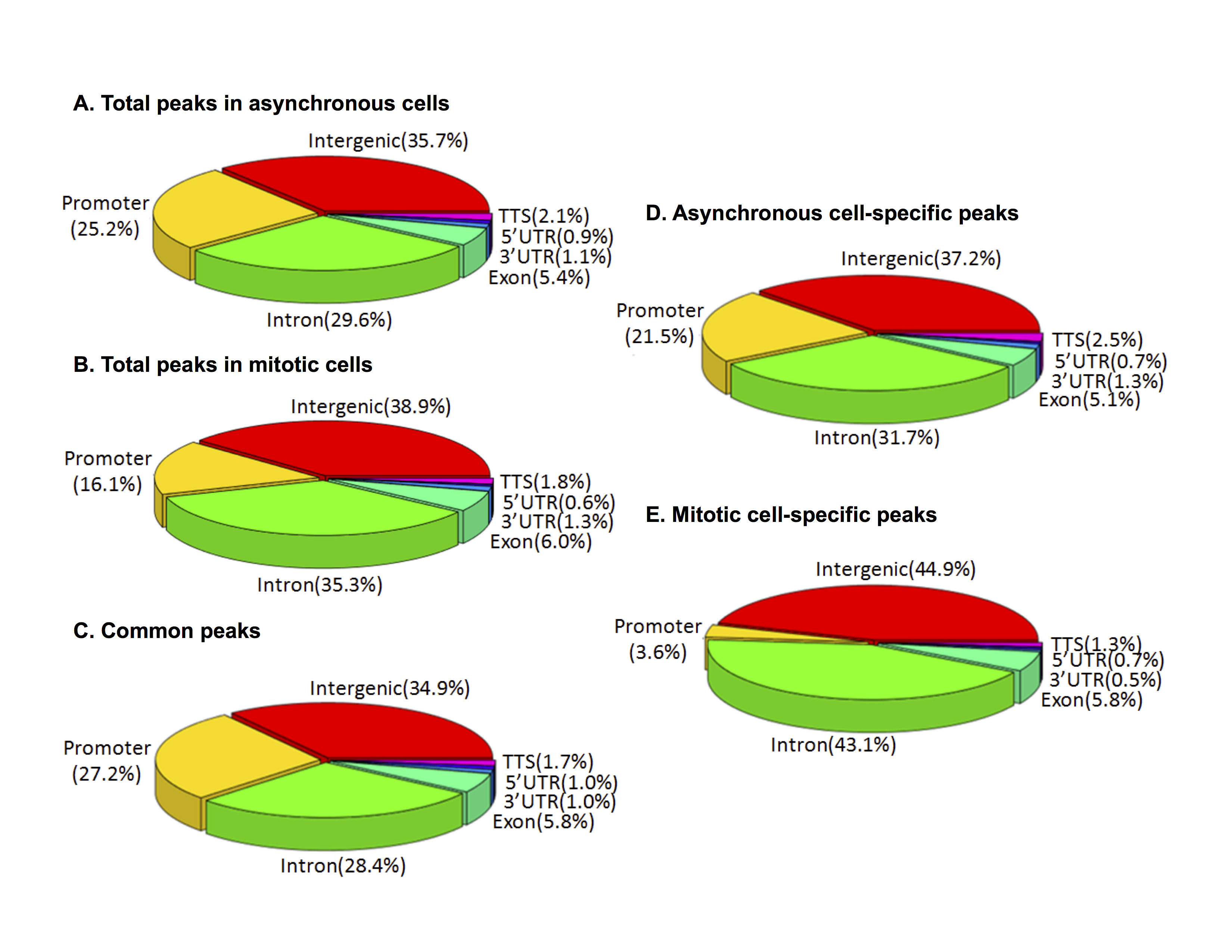

Supplement: Figure S6 — Pie charts illustrating the genomic distribution of RBPJ occupancy, as determined by gene annotation. (A) Distribution of total RBPJ occupancy on chromatin of asynchronous cells. (B) Distribution of total RBPJ occupancy on mitotic chromatin. (C) Distribution of RBPJ occupancy common to asynchronous and mitotic cells. (D) Distribution of RBPJ occupancy unique to asynchronous cells. (E) Distribution of RBPJ occupancy unique to mitotic cells. (TIFF) [file pgen.1004204.s006.tif]

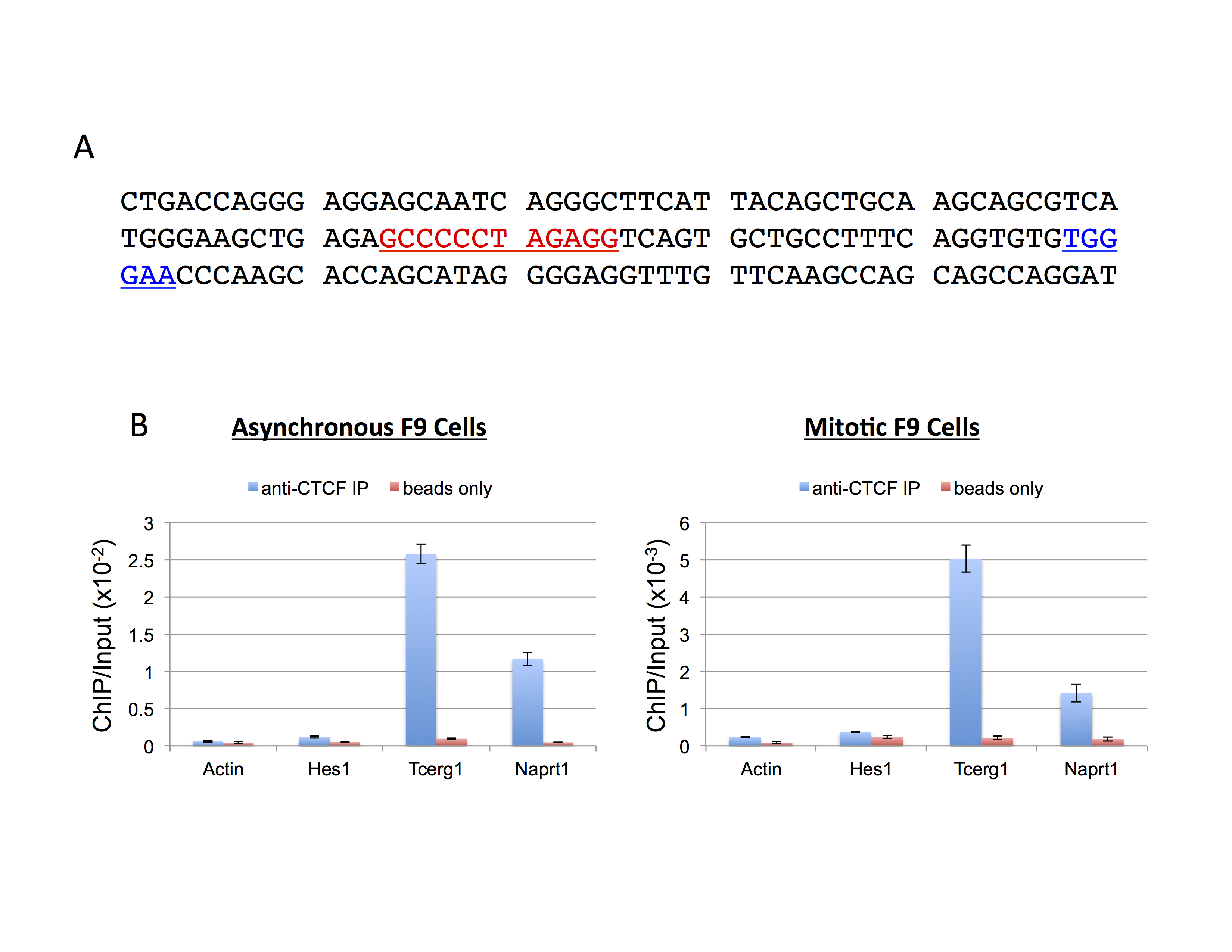

Supplement: Figure S7 — Association of the CTCF protein with the Naprt1 and Tcerg1 promoters, as revealed by ChIP-qPCR. (A) Naprt1 contains both CTCF- and RBPJ-binding motifs (shown in red and blue, respectively), with the CTCF-binding motif positioned at the center of the RBPJ ChIP sequencing peak. (B) Anti-CTCF ChIP-qPCR demonstrating that CTCF binds to the Naprt1 and Tcerg1 promoters, but not to Hes1 or actin, in both asynchronous and mitotic F9 cells. (TIFF) [file pgen.1004204.s007.tif]

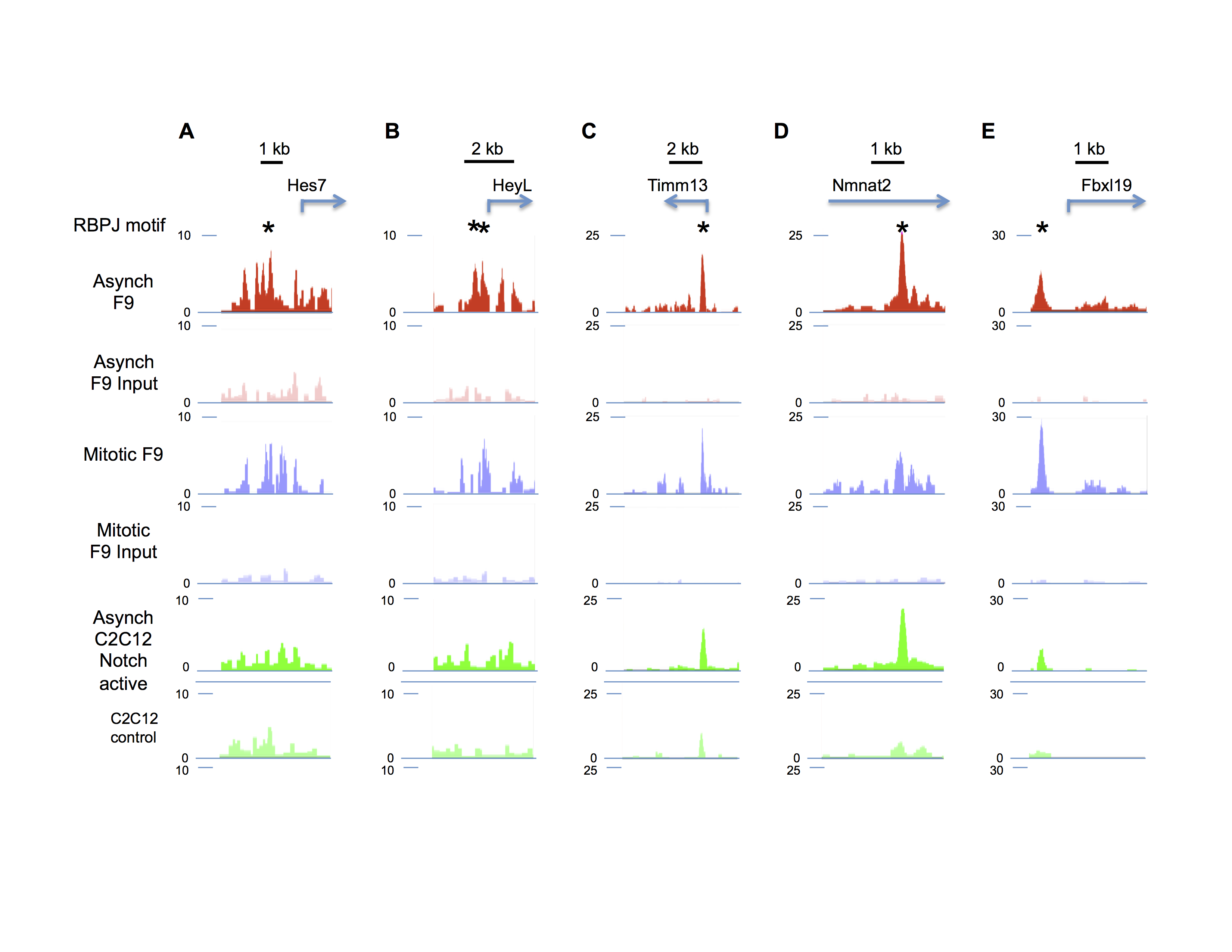

Supplement: Figure S8 — RBPJ binds to Notch responsive genes in asynchronous and mitotic F9 cells. Screen shots from the UCSC Genome Browser revealing RBPJ occupancy at Notch responsive genes. The position of the RBPJ-binding motif within each peak is indicated with an asterisk. The transcription factors Hes7 (A) and HeyL (B) are representative Notch-target genes of the Hes and Hey families. Timm13 (C), Nmnat2 (D) and Fbxl19 (E) are from Castel and Mourikis et al. [51]. Coordinates of the regions shown are (A) chr11: 68,930,148-68,935,117, (B) chr4:122,908,000-122,912,999, (C) chr10:80,359,119-80,366,062, (D) chr1:154,936,858-154,940,532, and (E) chr7:134,889,218-134,893,011. (TIFF) [file pgen.1004204.s008.tif]

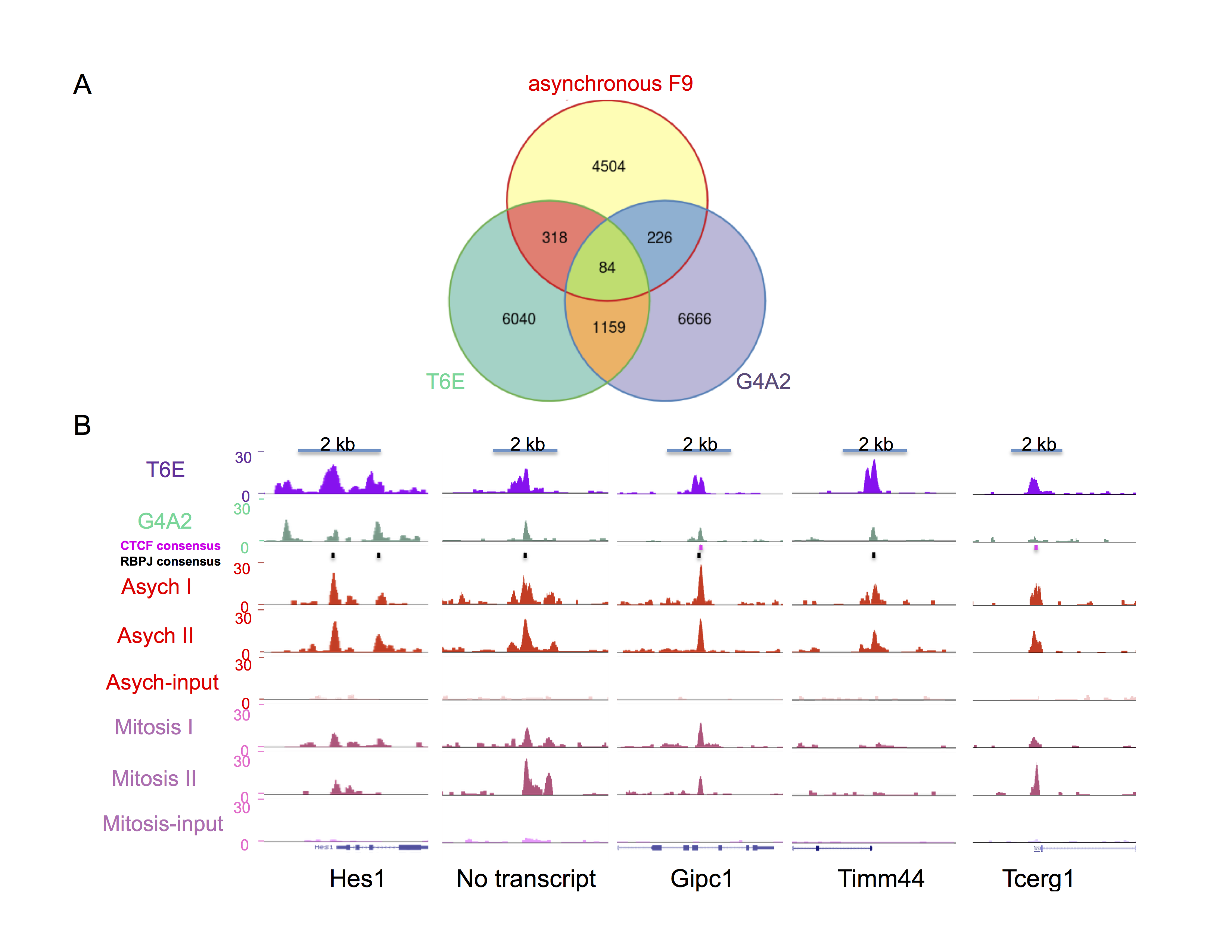

Supplement: Figure S9 — Comparisons of RBPJ ChIP-seq results obtained from F9 cells to those of the TLL cell lines, T6E and G4A2. (A) Venn diagram illustrating the overlap of RBPJ occupancy sites in F9 cells (this study) and in T6E and G4A2 cells [37]. (B) Screen shots taken from the UCSF Genome Browser showing side-by-side comparison of RBPJ occupancy at five regions. Also included are duplicates from asynchronous and mitotic F9 cells as well as input controls. The RBPJ- and CTCF-binding motifs are marked with black and pink squares, respectively. The coordinates of these five loci from left to right are (1) chr16:30,055,655-30,076,174, (2) chr14:76,549,884-76,555,073, (3) chr8:86,183,791-86,188,980, (4) chr8:4,273,375-4,278,564 and (5) chr18:42,668,310-42,675,184. Region 1, 4 and 5 contain the promoters of Hes1, Timm44 and Tcerg1, respectively. No transcript is found associated with region 2, and the peaks shown in region 3 lay in an intron of Gipc1. (TIFF) [file pgen.1004204.s009.tif]
