# Supplementary material for: RBPJ, the Major Transcriptional Effector of Notch Signaling, Remains Associated with Chromatin throughout Mitosis, Suggesting a Role in Mitotic Bookmarking
Source: PLoS Genet. 2014 Mar 6;10(3):e1004204. doi: 10.1371/journal.pgen.1004204 (PMC3945225; doi:10.1371/journal.pgen.1004204)
Supplement: Table S6 — Primers used in real-time PCR. (DOCX) [file pgen.1004204.s015.docx]

**Table S6:Primers used in real-time PCR**

| Primer | Sequence (5’ to 3’) |
| --- | --- |
| Hes1_Fw | TCCTCCCATTGGCTGAAAGTT |
| Hes1_Rv | ATATCTGGGACTGCACGCGAAC |
| Naprt1_Fw | GCACCAAAGGCAAAACAACT |
| Naprt1_Rv | CTCTAGGGGGCTCTCAGCTT |
| Tcerg1_Fw | GGAACTTGGGAGGACCTAGC |
| Tcerg1_Rv | AGTTGATTGGCCGCTTAGAG |
| Actin _Fw | GACCAGGCCGTATATGGAGA |
| Actin _Rv | AGCAGTCTGCAAAGCAGTGA |
| chr2_green_F | CCCAAACAGCTGGCCTACTA |
| chr2_green_R | TAGAGAGCGCGATTCGTCTT |
| chr2_blue_F | GCTGGGCTTCAGTTATTTGG |
| chr2_blue_R | GCGGCTTGTATTCTGGTACTG |
| chr4_blue_F | CAGGGAACCTTTGTCATTCAA |
| chr4_blue_R | CTCTGGAGGCAAAGGAGAAA |
| chr4_green_F | CAGTTCTCCCCACCTCTCTC |
| chr4_green_R | GATTAGTTCGGGTCCCCTGT |
| chrX_gray-F | GAGGAAGTGGAGCCGATG |
| chrX_gray-R | CCGTATGCCTGGATGATTTA |
| chrX_blue-F | TGTGCTGAGCTCCCTACAGA |
| chrX_blue-R | GCCGCAACGGAAGTTTATAG |
| chrX_green-F | GCCTCTCTCTTTCCGAGCTG |
| chrX_green-R | TGTACACGCTGGGTACAAGG |
